# Proto-Biosignatures and Planetary Geochemical Metabolism: A Thermodynamic Screening Model of Prebiotic Geochemical Organization

**DOI:** 10.3390/life16081312

**Published:** 2026-08-10

**Authors:** Sebastiano Ettore Spoto

**Affiliations:** Dipartimento di Scienze della Terra, Università degli Studi di Firenze, 50121 Firenze, Italy; sebastianoettore.spoto@unifi.it

**Keywords:** astrobiology, astrobiological exploration, geochemistry, proto-biosignatures, biosignatures, chemical disequilibrium, mineral catalysis, reaction networks, ocean worlds, exoplanets

## Abstract

Astrobiological observations usually return atmospheric, mineralogical, molecular, isotopic, or textural states rather than organisms. The interval between environmental habitability and confirmed life detection is therefore an evidential problem. This article develops *planetary geochemical metabolism* (PGM) as a scale-explicit description of abiotic water–rock–fluid reactions, including atmospheric or ice-shell boundary conditions where relevant, that sustain redox disequilibria, catalytic mineral interfaces, prebiotic molecular fluxes, and preservable mineral products. *Proto-biosignatures* are defined as contextual, non-diagnostic signatures of this interval: signals that do not demonstrate life, but increase the plausibility of prebiotic network organization relative to low-organization abiotic chemistry. A nondimensional Geochemical Metabolic Potential, $\Phi_{\mathrm{PGM}}$, is formalized as a target-specific screening index describing redox exergy, catalytic-interface density, reaction-network closure, environmental cycling efficacy, and preservation potential. The index is not calibrated as a probability of life. A reproducible Latin-hypercube experiment across $M_{\mathrm{sim}}=5000$ synthetic environments examines internal model behavior rather than ranking planets. Higher $\Phi_{\mathrm{PGM}}$ values mark hypotheses to be tested under declared scale, uncertainty, and observability constraints. Applications to early Earth, Noachian Mars, ocean worlds, and terrestrial exoplanets illustrate that habitability, prebiotic organization, biological inference, and preservation are distinct quantities.

## 1. Introduction

The astrobiological search for life is increasingly constrained by an evidential asymmetry. The observations most likely to be obtained from other worlds are not observations of organisms, but observations of atmospheric, mineralogical, molecular, isotopic, or textural states that must be interpreted against incomplete models of abiotic chemistry. Modern biosignature science has therefore moved away from the expectation of a single decisive marker and toward contextual assessment, likelihood comparison, false-positive analysis, and confidence scales for evidence of life [1,2,3,4,5,6]. This shift is necessary, but it leaves a more specific problem unresolved: how should one interpret environments that are not biologically diagnostic, yet are not low-organization abiotic systems because they display energy flow, mineral catalysis, recurrence, and molecular organization plausibly relevant to life’s emergence?

In practical terms, the problem addressed here is not how to infer life from ambiguous geochemical signals. It is how to evaluate whether such signals record a physically organized prebiotic regime rather than low-organization abiotic chemistry. The model asks a narrower question: does a target contain enough redox work, mineral mediation, reaction-network coupling, environmental recurrence, and archive quality to make non-biological prebiotic organization plausible and testable?

The distinction between habitability and inhabitedness is therefore not sufficient for early planetary environments. Liquid water, chemical energy, and the presence of carbon, hydrogen, nitrogen, oxygen, phosphorus, sulfur, and catalytic metals are necessary boundary conditions for many prebiotic and life-detection scenarios, but they do not by themselves define a path from geochemistry to biological inference [7,8,9,10]. A planet may be habitable without having crossed into biology, and conversely a planet may preserve traces of life long after surface conditions have become hostile. This asymmetry is evident in the contrast between Mars, where sedimentary and diagenetic archives may retain ancient traces despite present surface hostility, and ocean worlds, where active water–rock chemistry may occur beneath thick ice shells but be difficult to preserve in an accessible archive and sample [11,12,13,14,15]. A model linking Earth science and life detection must therefore keep habitability, prebiotic chemical organization, biological activity, and preservation analytically separate while allowing them to interact.

A recently published Mars-specific formulation has made the same separation in a target-restricted form by treating prebiotic synthesis, metabolic plausibility, and biosignature preservation as physically coupled but partially displaced astrobiological windows along mineral–organic–aqueous reactor–filter trajectories [16]. The present article generalizes that logic beyond Mars: it does not assume that synthesis, energy transduction, and preservation reach their maxima in the same place or at the same time, and it asks how such partial decoupling should be treated in a cross-target screening model.

This article develops that separation through *planetary geochemical metabolism* (PGM). The term denotes a non-biological analogue of metabolism in planetary environmental contexts: not a literal organismal metabolism, but a structured network of geochemical reactions that channels free energy through matter, couples electron donors and acceptors across mineral–water–fluid interfaces, incorporates atmospheric or ice-shell boundary conditions when they are part of the target system, forms and destroys organic compounds, and produces mineralogical memory. The adjective planetary does not imply a single global average; in this model, PGM is evaluated over declared environmental control volumes and timescales and is then interpreted within the geological context of a planetary body. The concept is motivated by the fact that many origin-of-life scenarios depend on geochemical engines rather than isolated reactions: serpentinization and alkaline hydrothermal vents, cyano-sulfidic surface chemistry, wet–dry cycling, mineral adsorption, catalysis by transition-metal sulfides, native metals, intermetallic alloys, oxides, phosphides, and mixed-valence surfaces, and autocatalytic chemical networks all require disequilibrium, interfaces, and recurrence [17,18,19,20,21,22,23,24,25,26]. “Metabolism” is used here only in a thermodynamic and network sense: a sustained transformation of free energy and chemical species before genetically encoded cellular metabolism.

On that basis, the article introduces *proto-biosignatures*. A proto-biosignature is not a claim of life, and it should not be treated as a weak biosignature. It is a measurable feature, or preferably a mutually constrained set of features, indicating that an environment occupied a regime in which geochemical disequilibrium, catalysis, cycling, and preservation jointly favored the emergence or persistence of prebiotic reaction networks. Candidate examples include redox–mineral–organic associations, non-random distributions of organics in catalytic mineral contexts, repeated mineral reaction fronts, low-amplitude but spatially coherent isotope patterns, or molecular-complexity patterns that exceed simple equilibrium expectations yet fall short of diagnostic biogenicity [27,28,29,30,31,32,33]. The category is intended to clarify the evidential ladder: it marks the process-level interval between geochemical habitability and biological evidence.

The article has four aims. First, it defines planetary geochemical metabolism and proto-biosignatures. Second, it formalizes a nondimensional Geochemical Metabolic Potential, $\Phi_{\mathrm{PGM}}$, from thermodynamic, kinetic, network, cycling, and preservation terms. Third, it tests the mathematical behavior of the index through a reproducible synthetic parameter-space experiment. Fourth, it translates the model into falsifiable expectations for early Earth, Mars, ocean worlds, and exoplanets without treating those targets as a numerical ranking. The contribution differs from existing biosignature catalogues because it does not merely list candidate signals; it specifies when the geochemical process capable of producing organized, non-biological prebiotic signals is itself plausible. PGM does not classify a signal as life-like; it supplies a process-level prior for the pre-biological interval. Each term is tied to measurable or modelable variables, and each assertion can be challenged by geochemical, laboratory, mission, or remote-sensing data [27,34,35,36,37,38].

The purpose is deliberately narrower than life detection and broader than ordinary habitability assessment. The article preserves the evidential value of non-diagnostic geochemical organization without converting that organization into a biological claim. This is the reason for introducing proto-biosignatures: they name a testable prebiotic interval, not a weaker form of biological evidence.

The working hypotheses are explicit. *H1*: prebiotic organization is favored when redox work, catalytic interfaces, reaction-network coupling, environmental recurrence, and preservation overlap at the same relevant scale. *H2*: candidate proto-biosignatures should increase evidence for $H_{\mathrm{pg}}$ relative to $H_{\mathrm{ab}}$ without collapsing into a biological claim. *H3*: low values of any required component should suppress the interpretation of $\Phi_{\mathrm{PGM}}$ even when one or more other components are high; in applications, this suppression is reported through $X_{\lim}$ and $f_{\mathrm{WL}}$, not by the scalar alone. These hypotheses are deliberately falsifiable because experiments or planetary samples can contradict the predicted co-occurrence of energy, catalysis, network closure, cycling, and preservation.

Table 1 positions the proposed model relative to existing life-detection approaches, including the Confidence of Life Detection (CoLD) scale and the Life Detection Knowledge Base (LDKB). The purpose is not to replace staged evidence scales, potential-biosignature catalogues, agnostic molecular metrics, or atmospheric disequilibrium analysis. PGM is complementary rather than hierarchically superior: it adds a process-level layer for environments that are more organized than low-organization abiotic chemistry but not biologically diagnostic.

## 2. Materials and Methods: Theoretical Model and Scope

### 2.1. Study Design and Evidential Target

This study is an original theoretical article. Its material is not a new planetary data set, but a formally specified geochemical model, a set of nondimensional observables, and a reproducible numerical demonstration designed to make the model’s behavior explicit. The argument proceeds in six steps. First, habitability, biogenicity, and prebiotic geochemical organization are separated as distinct evidential categories. Second, planetary geochemical metabolism is defined as an open-system reaction-network process rather than as a metaphor for biological metabolism. Third, the principal variables are fixed in a single notation table so that thermodynamic, kinetic, network, archival, and probabilistic terms cannot be confused. Fourth, each component of the Geochemical Metabolic Potential is tied to a physical or chemical quantity: redox exergy, catalytic surface density, reaction-network closure, environmental cycling, and signal preservation. Fifth, a synthetic Latin-hypercube experiment explores the five-dimensional parameter space without claiming empirical planetary rankings. Sixth, the resulting model is used to generate falsifiable predictions for early Earth, Mars, ocean worlds, and exoplanets.

The evidential target is therefore not life detection in the strict sense. The target is the intermediate state in which geochemical systems are sufficiently powered, catalyzed, connected, recurrent, and preserved to produce observations that deserve more weight than ordinary abiotic background chemistry but less weight than diagnostic biosignatures. This distinction is essential for avoiding both overclaiming and underusing astrobiologically valuable data.

### 2.2. Construction Rules and Boundary Conditions

The model is built under five rules. First, every PGM component is nondimensional and bounded in [0,1], so that no component can dominate solely because it is measured in larger units. Second, each component is monotonic in its intended physical quantity: increasing accessible redox work, catalytic surface efficacy, network closure, cycling efficacy, or preservation cannot lower the corresponding score while all else is held constant. Third, $\Phi_{\mathrm{PGM}}$ is not calibrated as a probability of life and is not a universal planetary ranking scale. It is a screening and hypothesis-generating index of detectable geochemical organization potential, and it is target-specific, control-volume-specific, timescale-specific, and signal-class-specific. A scalar value is not admissible without the full component vector, uncertainty ranges, environmental assumptions, weights, regularization constant, and limiting component. Fourth, weights are context-dependent and must be declared; they are not universal constants of abiogenesis. Fifth, every proto-biosignature claim must be evaluated against at least two non-biological alternatives: low-organization abiotic chemistry and non-biological prebiotic geochemical organization. These rules make the theory restrictive enough to be tested and cautious enough to avoid treating geochemical promise as biological evidence.

These rules also define exclusion criteria. A single organic molecule without geological context, a hydrated mineral deposit without energy or catalytic information, an atmospheric disequilibrium without source–sink analysis, or a complex mass spectrum with unresolved contamination history is not sufficient to define a proto-biosignature in the present sense. Such observations may be important inputs, but they become proto-biosignature evidence only when they are placed within a constrained environmental model that can compare low-organization abiotic chemistry with prebiotic geochemical organization [3,5,27,38,39].

### 2.3. Scale, Control Volume, and Observational Support

A central point is that PGM is not scale-free. Redox gradients, native metal grains, alloy inclusions, sulfates, sulfides, organic films, fluid pathways, and preservation hosts may coexist at micrometre to metre scales while being erased by bulk averaging. Conversely, regional or planetary statements may be useful only after many local control volumes have been aggregated with an explicit upscaling rule. The model is therefore written as

(1) $$\;\Phi_{\mathrm{PGM}}=\Phi_{\mathrm{PGM}}\left(V_{c},\Delta t_{c},S_{\mathrm{obs}},C_{\mathrm{env}}\right),$$

where $V_{c}$ is the declared environmental control volume, $\Delta t_{c}$ is the temporal window over which reaction, alteration, cycling, and preservation are evaluated, $S_{\mathrm{obs}}$ denotes the observational or analytical support of the measurements, and $C_{\mathrm{env}}$ is the geological context. Two scores are comparable only when these four elements, together with the signal class, weights, reference scales, and missing-data policy, are declared. A centimetre-scale Martian mudstone, a micrometre-scale serpentinized mineral domain, an active hydrothermal vent chimney, an ice-covered ocean-world plume source, and a remotely sensed exoplanet atmosphere are therefore not different samples of one universal scalar; they are different implementations of Equation (1).

Table 2 gives the operational scale ladder used in this article. The purpose is not to prescribe fixed length scales, but to prevent the most common error: interpreting a bulk or remote score as if it preserved the microscale coexistence of oxidized and reduced domains. Native metals and intermetallic compounds in ultramafic and subduction-related rocks, including Fe–Ni alloys and platinum-group-element-bearing phases, illustrate why scale matters: reduced phases can occur close to more oxidized mineral microdomains, and their interpretation depends on texture, host, alteration history, and analytical resolution [40].

### 2.4. Habitability, Biogenicity, and the Missing Intermediate Category

We use *habitability* to denote the capacity of an environment to support life as constrained by solvent availability, temperature, pressure, chemical energy, bioessential elements, and physicochemical stability. We use *biogenicity* to denote the posterior plausibility that an observed signal was produced by life. These terms should not be collapsed. Habitability is an environmental property; biogenicity is an evidential inference. The literature on exoplanet biosignatures has repeatedly emphasized this distinction through environmental context, atmospheric false positives, and likelihood-based assessment [1,2,39,41,42]. The same distinction is required for rocks, sediments, hydrothermal systems, and ocean-world plume materials.

The missing intermediate category is *prebiotic geochemical organization*. Such organization is more specific than habitability because it requires free-energy transduction, spatial interfaces, chemical coupling, and recurrence. Yet it is less specific than life because it need not include heredity, cellularity, translation, or sustained Darwinian evolution. The category is not merely philosophical. It affects sampling strategy. A rover, lander, plume sampler, or telescope should not only ask whether a target is habitable or whether a signal is biological; it should also ask whether the target contains evidence for geochemical processes that can plausibly generate proto-metabolic networks and preserve their products [10,11,12,27].

### 2.5. Planetary Geochemical Metabolism

We define *planetary geochemical metabolism* (PGM) as the coupled set of abiotic water–rock–fluid reactions through which a planetary body converts environmental disequilibria into persistent fluxes of matter, charge, heat, and mineralogical information, with atmospheric, ice-shell, or gas-phase boundary conditions included where relevant.

The concept is most transparent in water–rock systems. Serpentinization couples the oxidation of ferrous iron in ultramafic minerals to the formation of molecular hydrogen, generating reducing power that can support abiotic organic synthesis and microbial metabolisms in modern analogues [19,21,22,43,44]. Alkaline hydrothermal vents add pH gradients, mineral membranes, transition-metal sulfides, native metals, alloys, oxides, phosphides, mixed-valence mineral surfaces, and natural compartmentalization, thereby bringing geochemistry into contact with principles that resemble the energetic architecture of early metabolism [17,18,20]. Surface environments, by contrast, can provide wet–dry cycling, photochemical activation, evaporation, freezing, impact processing, and concentration-dilution sequences that may drive condensation and selection-like behavior in complex mixtures [9,23,24,45].

PGM therefore differs from conventional habitability metrics. Habitability often treats energy, water, and elements as inventory variables. PGM treats them as *flux variables* embedded in networks. A planet with abundant water but weak redox gradients may have low PGM. A planet with strong disequilibrium but no preservation pathway may have high chemical activity and low recoverable evidence. A planet with ancient sediments, clays, carbonates, sulfates, and redox interfaces may have strong preservation even if its current geochemical energy flow is low [11,12,46,47]. This formalism thus allows active and archival worlds to be compared without forcing them into the same evidential category.

### 2.6. Planetary Transferability and Hydrothermal Diversity

Earth analogues are necessary but conditional. They are not constants of planetary chemistry. Water–rock reaction pathways depend on protolith history, melt differentiation, volatile inventories, crust–mantle–atmosphere exchange, oxidation state, impact history, heat flow, permeability, and the availability of electron donors and acceptors. A basaltic oceanic crust analogue, a komatiitic or ultramafic Hadean setting, a Martian basaltic sedimentary system, and an icy moon seafloor may share water–rock reaction motifs while differing in metal speciation, pH, redox buffering, sulfur chemistry, phosphorus mobility, and archive formation. For that reason, terrestrial measurements enter PGM as conditional priors and laboratory constraints, not as universal planetary defaults.

Hydrothermal systems also should not be collapsed into one category. Table 3 distinguishes the principal classes relevant to this article. The same five PGM terms apply to each class, but their physical meaning changes with fluid source, thermal regime, mineralogy, permeability, residence time, redox structure, and preservation setting.

### 2.7. Proto-Biosignatures

A *proto-biosignature* is defined here as an observable pattern b that satisfies three conditions. First, b is more probable under a prebiotic geochemical-organization hypothesis than under a low-organization abiotic null. Second, b is not by itself sufficient to infer extant or extinct life. Third, b is interpretable only in environmental context, especially the coexistence of energy flux, catalysis, network connectivity, cycling, and preservation. This definition follows the logic of biosignature threshold analysis but assigns a distinct evidential role to prebiotic or proto-metabolic states [3,5,27,37,38].

The category is useful because many candidate observations are scientifically important even when they cannot support a life claim. Redox–organic–mineral associations in a fine-grained Martian mudstone, complex organics in an Enceladus plume, or high molecular assembly scores in a non-terrestrial sample would all be overinterpreted if called biosignatures without context. Yet they would be underinterpreted if treated as merely abiotic detections. Proto-biosignature language provides an intermediate evidential status: a signal that a system may have approached, sustained, or archived the geochemical preconditions for biological emergence [28,29,47,48,49,50].

## 3. Notation and Variables

Because the proposed theory combines thermodynamics, kinetics, network theory, and evidence assessment, notation must be explicit. Table 4 defines every principal variable used below. Italic symbols denote scalars, bold italic symbols denote vectors, upright bold uppercase symbols denote matrices, calligraphic uppercase symbols denote potentials, sets, or likelihood quantities, and Greek uppercase symbols denote nondimensional PGM components. No state variable and no PGM component share the same symbol.

## 4. Thermodynamic Basis of Planetary Geochemical Metabolism

### 4.1. Reaction Networks and Chemical Affinity

Consider an open planetary microenvironment, such as a hydrothermal pore, a wet–dry evaporitic basin, a mineral fracture, a sediment–water interface, or an ocean-world seafloor. Let $n\in R_{\geq 0}^{N_{s}}$ be the vector of species amounts and let $S\in R^{N_{s}\times N_{r}}$ be the stoichiometric matrix. The open-system mass balance is

(2) $$\;\frac{dn}{dt}=SJ+q_{\mathrm{in}}-q_{\mathrm{out}}+q_{\mathrm{phase}},$$

where J is the vector of net reaction fluxes, $q_{\mathrm{in}}$ and $q_{\mathrm{out}}$ are transport terms, and $q_{\mathrm{phase}}$ includes precipitation, dissolution, adsorption, desorption, and gas exchange. Equation (2) is general enough to include hydrothermal vent systems, sedimentary pore waters, atmospheric photochemical columns, and icy-ocean plume source regions, provided the relevant fluxes and phases are specified [34,51,52,53].

For species *i*, the chemical potential is

(3) $$\mu_{i}=\mu_{i}^{\circ }\left(T,P\right)+RT\ln a_{i},$$

where $\mu_{i}^{\circ }$ is the standard chemical potential at temperature *T* and pressure *P*, *R* is the gas constant, and $a_{i}$ is the activity. The Gibbs free energy change of reaction *r* is

(4) $$\Delta_{r}G=\sum_{i=1}^{N_{s}}S_{ir}\mu_{i}=\Delta_{r}G^{\circ }+RT\ln Q_{r},$$

where $Q_{r}=\prod_{i}a_{i}^{S_{ir}}$ is the reaction quotient. We define the chemical affinity as

(5) $$A_{r}=-\Delta_{r}G.$$

Positive affinity denotes a thermodynamic drive in the forward stoichiometric direction. This sign convention is retained throughout the paper. It is crucial because PGM is not defined by the mere existence of reactions, but by the existence of reactions whose affinities can be continuously maintained by planetary boundary conditions [51,52,54].

For an isothermal closed subsystem without external mass transport, the time derivative of Gibbs free energy is obtained by combining ${\dot{n}}_{i}=\sum_{r}S_{ir}J_{r}$ with Equation (4):

(6) $$\dot{G}=\sum_{i=1}^{N_{s}}\mu_{i}{\dot{n}}_{i}=\sum_{r=1}^{N_{r}}J_{r}\sum_{i=1}^{N_{s}}S_{ir}\mu_{i}=\sum_{r=1}^{N_{r}}J_{r}\Delta_{r}G=-\sum_{r=1}^{N_{r}}A_{r}J_{r}\leq 0.$$

The corresponding internal entropy-production rate is

(7) $${\dot{\Sigma }}_{\mathrm{int}}=\frac{1}{T}\sum_{r=1}^{N_{r}}A_{r}J_{r}\geq 0.$$

Equation (7) does not imply that life maximizes entropy production, nor that high dissipation alone produces biology. It states the more modest and necessary point that any proto-metabolic geochemical network must be embedded in positive entropy production, because it exists only while externally maintained gradients drive matter through reaction channels [51,52,54]. The inequality assumes thermodynamically consistent flux-force relations, or spontaneous relaxation toward equilibrium, under the stated reaction orientations; reversible pairs and coupled redox half-reactions should not be double counted as independent dissipative pathways. The novelty of PGM lies in coupling this thermodynamic condition to catalysis, closure, cycling, and preservation.

### 4.2. Redox Free Energy and the Nernst Relation

Many plausible origin-of-life and life-detection contexts depend on redox disequilibria: H_2_/CO_2_, Fe^2+^/Fe^3+^, S^2−^/SO_4_^2−^, CH_4_/CO_2_, and oxidant-reductant pairs produced by photochemistry, volcanism, serpentinization, radiolysis, or atmospheric escape [21,22,34,41,48]. For a redox reaction *r* transferring $z_{r}$ moles of electrons per mole of reaction, electrochemical work gives

(8) $$\;\Delta_{r}G=-z_{r}FE_{r},$$

where *F* is Faraday’s constant and $E_{r}$ is the cell potential. Combining Equations (4) and (8) yields the Nernst equation,

(9) $$E_{r}=E_{r}^{\circ }-\frac{RT}{z_{r}F}\ln Q_{r}.$$

This relation makes explicit why redox disequilibrium is a natural quantitative bridge between geochemistry and metabolism. The same physical quantity, $\Delta_{r}G$, appears as chemical affinity, available electrochemical work, and a constraint on reaction directionality. Geochemical systems that maintain large $E_{r}$ values over long times and across mineral interfaces are not alive, but they supply the energetic architecture from which proto-metabolic chemistry can draw [17,19,20,53].

For illustration, the reduction of carbon dioxide by hydrogen to methane,

(10) $${\mathrm{CO}}_{2}+4H_{2}\to {\mathrm{CH}}_{4}+2H_{2}O,$$

is central to both hydrothermal origin-of-life discussions and modern microbial energy metabolism. The significance of Equation (10) is not that abiotic methane is a biosignature. It is not. The significance is that the H_2_/CO_2_ couple links serpentinization, carbon reduction, mineral catalysis, and biological methanogenesis through a common redox thermodynamic structure [18,21,22,43,44]. A proto-biosignature approach therefore evaluates methane, organics, carbonates, magnetite, sulfides, and contextual mineralogy jointly rather than treating any single species as diagnostic.

### 4.3. Exergy Flux as a Necessary but Insufficient Condition

We define the local redox power term as

(11) $$P_{\mathrm{ex}}=\int_{V}\sum_{r\in R_{\mathrm{red}}}{\left[A_{r}\;\varrho_{r}\left(y,t\right)\right]}_{+}\;dV,$$

where V is the relevant control volume, $R_{\mathrm{red}}$ is the subset of redox reactions, $\varrho_{r}\left(y,t\right)$ is the local volumetric net reaction-rate density in mol m^−3^ s^−1^, and ${\left[u\right]}_{+}=\max\left(0,u\right)$. The dimensional check is explicit: $A_{r}\varrho_{r}dV$ has units of J s^−1^. The volume-integrated flux used elsewhere is $J_{r}=\int_{V}\varrho_{r}\left(y,t\right)\;dV$ when a single control-volume flux is required. The positive-part operator counts only locally dissipative redox work in the specified reaction orientation; it does not count an affinity that is inaccessible to the actual flux field. A reaction with large affinity but negligible volumetric flux cannot structure chemistry. This quantity is not the total dissipative power of an open system and is not the fraction of work captured by any specified synthetic pathway. Applications must still evaluate permeability, transport, phase transfer, kinetic accessibility, catalytic coupling, and passivation. If a particular pathway is modeled, a captured-power term can be written as $P_{\mathrm{cap}}=\zeta_{\mathrm{cap}}P_{\mathrm{ex}}$, with $\zeta_{\mathrm{cap}}\in \left[0,1\right]$ specified independently. This distinction is important for astrobiology: a world may contain theoretical redox energy, but without permeability, fluid circulation, catalytic surfaces, or appropriate kinetics the energy may not be chemically accessible [14,19,48,53].

The first nondimensional component of PGM is therefore

(12) $$\Theta_{\mathrm{red}}=1-\exp\left(-\frac{P_{\mathrm{ex}}\tau_{\mathrm{obs}}}{E_{\mathrm{ref}}}\right),$$

where $\tau_{\mathrm{obs}}$ is the observation or residence timescale and $E_{\mathrm{ref}}$ is a reference energy required to sustain a specified amount of chemical transformation in the modeled environment. Equation (12) maps redox-work availability into [0, 1] without implying a universal threshold. Other chemical, photochemical, pH, evaporative, or thermal disequilibria can be added as separate drive terms in target-specific implementations, but they are not silently folded into $\Theta_{\mathrm{red}}$. The reference energy is not a fitted life-detection threshold: it must be fixed before scoring for the declared control volume, signal class, and observation or residence timescale, and sensitivity to $E_{\mathrm{ref}}$ should be reported. The reference energy must be chosen by context: a hydrothermal vent pore, a Martian mudstone, an Enceladus ocean plume source, and an exoplanet atmosphere have different control volumes, residence times, and observables [1,10,34].

## 5. From Geochemical Flux to Proto-Metabolic Organization

### 5.1. Catalytic Mineral Interfaces

Thermodynamic drive alone is insufficient because many prebiotic transformations face kinetic barriers. Mineral surfaces can adsorb reactants, concentrate dilute species, stabilize transition states, orient molecules, mediate electron transfer, generate pH and redox microenvironments, and preserve products [17,21,33,55,56,57]. We therefore define the catalytic-interface density score as

(13) $$\Lambda_{\mathrm{cat}}=1-\exp\left[-\frac{1}{A_{\mathrm{ref}}}\sum_{m=1}^{N_{m}}\sum_{f=1}^{N_{f}}\pi_{f}\kappa_{m,f}A_{\mathrm{surf},m}\right],$$

where $N_{m}$ is the number of mineral classes, $N_{f}$ is the number of declared reaction families, $A_{\mathrm{surf},m}$ is reactive surface area, $\kappa_{m,f}\in \left[0,1\right]$ is the reaction-family-conditioned catalytic efficacy of mineral class *m* for family *f*, $\pi_{f}$ is a declared reaction-family weight with $\sum_{f}\pi_{f}=1$, and $A_{\mathrm{ref}}$ is a reference surface area for the modeled environment.

For clarity, this article uses mineralogical terminology in a restricted operational sense. “Catalytic mineral interface” denotes a reactive surface exposed to fluid and relevant to a declared reaction family; it is not the same as bulk mineral abundance. “Redox-sensitive mineral assemblage” denotes a paragenetic association recording oxidation-state gradients, redox fronts, or juxtaposed reduced and oxidized microdomains. “Transition-metal catalyst” includes Fe–Ni sulfides when justified, but also native metals, intermetallic alloys, phosphides, oxides, oxyhydroxides, spinels, mixed-valence phases, Cu-, Zn-, Co-bearing phases, and platinum-group-element-bearing phases where a reaction family and mechanism are declared. Table 5 summarizes the terminology used below.

The parameter $\kappa_{m,f}$ is not a mineralogical popularity score. It should be estimated from measured or modeled rate enhancements for declared reaction families: carbon reduction on transition-metal sulfides, native metal or alloy surfaces, phosphate activation, nucleotide adsorption and oligomerization on clays, sulfur redox cycling, or mixed-valence transition-metal catalysis in reverse tricarboxylic-acid-like sequences [21,23,56,57,58,59]. A mineral assemblage can therefore score highly in one prebiotic context and poorly in another. Operational estimates of $\kappa_{m,f}$ should state the reaction family, pH, temperature, reactive surface area, passivation state, competitive adsorption, grain size, and fluid accessibility. This context dependence is a strength of the model because proto-biosignature interpretation should depend on the reaction family under consideration.

### 5.2. Reaction-Network Closure and Connectivity

A geochemical environment becomes proto-metabolically interesting only when reactions are coupled into chemically feasible networks. For quantitative use, the allowed reaction set must be specified before the score is computed: feedstock species, activities, temperature, pressure, directionality, thermodynamic sign, kinetic accessibility, and transport renewal should all be declared. A reaction can contribute to network structure only if it is mass-balanced and feasible under the stated conditions, or if the uncertainty model assigns it a stated probability of feasibility.

Let G be a projected graph obtained from this filtered reaction set, with chemical species as nodes. Edges may be binary in a first screening calculation, but quantitative applications should weight them by estimated flux, thermodynamic feasibility, or reaction-family confidence. Let $\lambda_{2}$ be the algebraic connectivity of the normalized graph Laplacian for the largest chemically relevant component. Let $C_{\mathrm{prod}}$ be the set of products reachable from feedstock species under the allowed reaction set, and let $C_{\mathrm{auto}}\subseteq C_{\mathrm{prod}}$ be the subset participating in catalytic or autocatalytic closure. We define the minimal operational descriptor

(14) $$\Omega_{\mathrm{net}}=\left(\frac{|C_{\mathrm{auto}}|}{|C_{\mathrm{prod}}|+\varepsilon_{\mathrm{card}}}\right)\left(\frac{\lambda_{2}}{\lambda_{2}+\lambda_{\mathrm{ref}}}\right),$$

where $\varepsilon_{\mathrm{card}}$ avoids division by zero and $\lambda_{\mathrm{ref}}$ is a reference connectivity scale fixed for the chosen graph construction. The first factor estimates closure; the second estimates integration. Both are needed. A sparse set of isolated reactions may make organics without producing network persistence, whereas a densely connected network without closure may still decay when external feedstocks fluctuate [25,26,52,60].

Equation (14) should be read as a replaceable first descriptor, not as a universal measure of prebiotic chemistry. Graph topology is not chemistry by itself. Quantitative applications should replace or complement it with stoichiometrically and thermodynamically constrained analyses, such as elementary flux modes, stoichiometric autocatalytic cores, reflexively autocatalytic and food-generated sets, deficiency theory, graph controllability, or stochastic thermodynamic cycle decompositions [25,26,52]. The essential point is that proto-biosignatures should not be inferred from molecular richness alone. Molecular diversity becomes astrobiologically meaningful when it is embedded in plausible pathways of production, coupling, feedback, and persistence [28,29,30,31].

A reported $\Omega_{\mathrm{net}}$ value is not interpretable unless the reaction set, feasibility thresholds, graph projection, edge weights, and normalization are reported. Empty reaction sets, graphs lacking a chemically relevant connected component, undefined $\lambda_{2}$, or empty $C_{\mathrm{prod}}$ are assigned $\Omega_{\mathrm{net}}=0$ unless a probabilistic feasibility model is explicitly specified. Table 6 gives the minimum protocol used by the present model. The protocol is intentionally conservative: uncertain reactions may be represented as distributions or excluded in a sensitivity analysis, but they should not be silently converted into unweighted graph edges.

### 5.3. Environmental Cycling and Kinetic Resonance

Prebiotic reactions often require environmental recurrence. Wet–dry cycles can concentrate solutes and drive condensation; freeze–thaw cycles can concentrate reactants in brines; hydrothermal circulation can alternate temperature, pH, redox state, and mineral contact; tidal flexing can renew ocean-world interfaces; and diurnal or seasonal photochemistry can supply reactive intermediates [9,14,23,24,45]. We model this effect by defining a Damköhler-like number for reaction *r*,

(15) $$\;D_{r}=\frac{\tau_{\mathrm{env}}}{\tau_{r}},$$

where $\tau_{\mathrm{env}}$ is the environmental cycling time and $\tau_{r}$ is the characteristic reaction time. Cycling is most effective when environmental recurrence is neither too fast for chemistry to proceed nor too slow to prevent degradation and dilution. A simple resonance-like score is

(16) $$\Psi_{\mathrm{cyc}}=\sum_{r=1}^{N_{r}}\alpha_{r}\exp\left[-\frac{{\left(\ln D_{r}\right)}^{2}}{2\sigma_{\mathrm{cyc}}^{2}}\right]C_{\mathrm{conc},r},$$

where $\sigma_{\mathrm{cyc}}$ controls tolerance around $D_{r}=1$, $C_{\mathrm{conc},r}\in \left[0,1\right]$ represents concentration enhancement without irreversible loss, and $\alpha_{r}\geq 0$ is a declared reaction-importance weight with $\sum_{r}\alpha_{r}=1$. The unweighted case $\alpha_{r}=1/N_{r}$ is acceptable only for synthetic demonstrations or when no reaction family is privileged by the model.

This expression is not meant to privilege surface cycling over hydrothermal cycling. Rather, it formalizes the claim that environmental periodicity matters only through its coupling to reaction timescales. If recurrence is absent, or if $\tau_{\mathrm{env}}$ or the relevant $\tau_{r}$ cannot be defined for the declared reaction family, $\Psi_{\mathrm{cyc}}$ is assigned zero for that family unless a stochastic or distributed-timescale model is specified. When cycling and reaction times are ranges rather than point values, Equation (16) should be evaluated over their uncertainty distributions rather than with a single nominal ratio. In a wet–dry pond, $\tau_{\mathrm{env}}$ may be hours to months; in a hydrothermal pore network, it may be the residence time of circulating fluids; in an icy moon, it may be controlled by tidal deformation, fracture transport, or plume ejection; in an atmosphere, it may be photochemical lifetime or vertical mixing time [24,34,45,48,49]. Proto-biosignatures should therefore be sought where environmental timing and reaction timing overlap.

### 5.4. Preservation as a Physical Filter

A signal cannot be detected if it is not preserved or sampled. Preservation is not a passive afterthought but a selective filter that shapes the evidential record. Organic molecules, isotopic gradients, redox-sensitive mineral assemblages, microtextures, and molecular-complexity distributions can be erased or overprinted by oxidation, radiation, hydrolysis, thermal metamorphism, diagenesis, impact processing, and contamination. Conversely, clays, silica, carbonates, sulfates, phosphates, and fine-grained sediments may enhance preservation under appropriate conditions [11,12,33,46,61].

We define preservation potential as

(17) $$\Xi_{\mathrm{pre}}=\chi_{\mathrm{host}}\exp\left[-\int_{0}^{t_{\mathrm{arch}}}k_{\mathrm{loss}}\left(t\right)\;dt\right],$$

where $\chi_{\mathrm{host}}\in \left[0,1\right]$ measures the suitability of the host phase for preserving the signal class, $t_{\mathrm{arch}}$ is archive duration, and $k_{\mathrm{loss}}\left(t\right)$ is the total signal-loss rate. We decompose

(18) $$k_{\mathrm{loss}}\left(t\right)=k_{\mathrm{ox}}\left(t\right)+k_{\mathrm{hyd}}\left(t\right)+k_{\mathrm{rad}}\left(t\right)+k_{\mathrm{therm}}\left(t\right)+k_{\mathrm{diag}}\left(t\right),$$

where the terms represent oxidative, hydrolytic, radiolytic, thermal, and diagenetic destruction or overprinting. This decomposition is particularly relevant to Mars, where ancient sedimentary archives may have high $\chi_{\mathrm{host}}$ but long exposure histories, and to ocean worlds, where plume materials may be fresh but source-region archives may be difficult to access [11,12,14,49,50].

$\Xi_{\mathrm{pre}}$ describes survival of a signal in an archive, not necessarily the probability that the archive can be reached or sampled. When physical accessibility, plume transport, sample-return feasibility, or contamination control dominates detectability, the access term should be reported separately or declared as an explicit sampling assumption rather than hidden inside preservation. This separation is especially important for subsurface-ocean targets, where source-region preservation and plume accessibility are distinct physical problems.

## 6. Model Formulation: The Geochemical Metabolic Potential

### 6.1. Definition

Origin potential and evidence potential should be kept separate. We first define an organization score that excludes preservation:

(19) $$\Phi_{\mathrm{org}}=\prod_{q=1}^{4}{\left[\varepsilon_{\Phi }+\left(1-\varepsilon_{\Phi }\right)X_{q}\right]}^{{\tilde{w}}_{q}},$$

where

(20) $$\left\{X_{1},X_{2},X_{3},X_{4}\right\}=\left\{\Theta_{\mathrm{red}},\Lambda_{\mathrm{cat}},\Omega_{\mathrm{net}},\Psi_{\mathrm{cyc}}\right\},$$

$0<\varepsilon_{\Phi }\ll 1$, ${\tilde{w}}_{q}\geq 0$, and $\sum_{q=1}^{4}{\tilde{w}}_{q}=1$. This score addresses whether a setting has energy, catalysis, network coupling, and recurrence consistent with prebiotic organization. It does not ask whether the products can be recovered.

For life-detection and sample-selection purposes, preservation must be included. We therefore define the Geochemical Metabolic Potential used in this article as the detectable proto-biosignature potential:

(21) $$\Phi_{\mathrm{PGM}}=\prod_{q=1}^{5}{\left[\varepsilon_{\Phi }+\left(1-\varepsilon_{\Phi }\right)X_{q}\right]}^{w_{q}},$$

where

(22) $$\left\{X_{1},X_{2},X_{3},X_{4},X_{5}\right\}=\left\{\Theta_{\mathrm{red}},\Lambda_{\mathrm{cat}},\Omega_{\mathrm{net}},\Psi_{\mathrm{cyc}},\Xi_{\mathrm{pre}}\right\},$$

$w_{q}\geq 0$ and $\sum_{q=1}^{5}w_{q}=1$. Equivalently,

(23) $$\;\ln\Phi_{\mathrm{PGM}}=\sum_{q=1}^{5}w_{q}\ln\left[\varepsilon_{\Phi }+\left(1-\varepsilon_{\Phi }\right)X_{q}\right].$$

The geometric form is chosen because PGM is limited by weak links. A chemically energetic environment with no catalytic interfaces, or a catalytic environment with no preservation, should not score highly as a proto-biosignature target. Arithmetic sums would obscure that constraint. Let $Y_{q}=\varepsilon_{\Phi }+\left(1-\varepsilon_{\Phi }\right)X_{q}$. Since the weights are non-negative and sum to unity, the weighted geometric mean obeys

(24) $$\;\min_{q:w_{q}>0}Y_{q}\leq \Phi_{\mathrm{PGM}}\leq \max_{q:w_{q}>0}Y_{q}.$$

Equality can occur when all positively weighted components share the same value, or when all positive weight is assigned to an extremal component; otherwise the inequalities are strict for non-identical positively weighted components. For reporting, the limiting component is

(25) $$\;X_{\lim}=\min_{q:w_{q}>0}X_{q},$$

and a target may be flagged as weak-link constrained by

(26) $$\;f_{\mathrm{WL}}=\left\{\begin{array}{cc} 1, & X_{\lim}<X_{\mathrm{thr}},\\ 0, & X_{\lim}\geq X_{\mathrm{thr}}, \end{array}\right.$$

where $X_{\mathrm{thr}}$ is a declared reporting threshold rather than a universal constant. The threshold must be fixed before scoring, reported with the sensitivity analysis, and never tuned post hoc to support or downgrade a target. If $f_{\mathrm{WL}}=1$, the scalar value should be treated as a diagnostic descriptor of the model construction, not as a priority score, unless the limiting component, threshold, uncertainty model, and physical reason for the weak link are stated. This flag is especially important because the regularized geometric mean can remain moderate when one component is very low and the remaining components are high.

The operational consequences of these restrictions are summarized in Table 7.

In the unregularized limit, if any component with $w_{q}>0$ approaches zero, $\Phi_{\mathrm{PGM}}$ also approaches zero. This limiting behavior is not an empirical law; it is a design choice that encodes the claim that proto-biosignature potential requires joint sufficiency rather than compensation by a single favorable variable. The regularization constant avoids numerical singularities but does not replace physical gating, declared thresholds, or the weak-link flag in Equation (26). The weights $w_{q}$ are not universal constants; they encode mission objective and signal class. For origin-of-life plausibility, $\Theta_{\mathrm{red}}$, $\Lambda_{\mathrm{cat}}$, $\Omega_{\mathrm{net}}$, and $\Psi_{\mathrm{cyc}}$ may dominate. For life-detection archive selection, $\Xi_{\mathrm{pre}}$ may require greater weight [11,12,27,37,38].

This formalism is intentionally conservative. A high $\Phi_{\mathrm{PGM}}$ is not a biosignature. It is a measure of detectable geochemical organization potential. It predicts that proto-biosignatures, if present, should cluster in environments where redox energy, catalytic mineral surfaces, reaction-network connectivity, environmental cycling, and preservation overlap. It also predicts that some environments with compelling habitability inventories may be poor proto-biosignature targets if one component is absent. This distinction may help explain why “follow the water” has gradually been supplemented by “follow the energy”, “follow the gradients”, “follow the organics”, and “follow the preservation” [3,10,11,12].

### 6.2. Operational Estimation

A practical implementation of Equation (21) would proceed in four steps. First, define the control volume and timescale relevant to the environment: a sedimentary bed, hydrothermal mound, ice-grain source region, plume, or atmospheric column. PGM is calculated for such declared volumes and times; planetary-context interpretation requires distributions over microenvironments or an explicit hierarchical aggregation model, not an unspecified planetary average. Second, compute or estimate reaction affinities and fluxes using geochemical speciation, thermodynamic databases, kinetic constraints, and transport models. Third, map mineralogy and reaction products into catalytic and preservation terms. Fourth, compare candidate feature vectors with abiotic and prebiotic models through likelihood ratios [14,34,52,53]. In compact form, the workflow is: control volume → signal class → reference scales → component distributions → uncertainty propagation → data partitioning.

For landed missions and sample return, the observables may include mineral assemblages, redox couples, organic distributions, isotopic ratios, grain-scale textures, Raman spectra, mass spectra, and stratigraphic context. For ocean-world plume sampling, the observables may be dissolved salts, molecular hydrogen, organics, phosphate, pH proxies, particle composition, and isotopic measurements. For exoplanets, the observables are mostly atmospheric gases and possibly surface spectral features; the PGM terms must therefore be marginalized over poorly constrained geology, interior redox state, volatile cycling, and stellar photochemistry [1,2,34,35,48,49,50]. In all cases, the score is a model-dependent hypothesis generator, not a direct measurement of life.

A reported application should therefore include a minimal audit trail: the control volume, the timescale, the assumed reaction set, the thermodynamic database or redox model, the mineral-catalytic mapping, the cycling model, the preservation model, the weights, the uncertainty range for each $X_{q}$, and the limiting component $\min_{q}X_{q}$. The limiting component should be stated alongside $\Phi_{\mathrm{PGM}}$, because the weakest term identifies the physical process most responsible for suppressing proto-biosignature potential. Without this audit trail, a numerical PGM value would be insufficiently reproducible. A reported value is admissible only with the component vector X, weights, $\varepsilon_{\Phi }$, control volume, timescale, signal class, uncertainty model, and limiting component.

Table 8 converts this audit trail into a minimum reporting standard. The standard is not a calibration database; it is a reproducibility requirement. Its purpose is to make clear which observations, assumptions, and negative controls produced each component distribution.

Incomplete observations should not be converted into optimistic component scores. Table 9 gives the conservative missing-data policy used by the model. The policy is intentionally stricter than a qualitative plausibility assessment because the most common failure mode of composite indices is to replace absent information with implicit expert confidence.

### 6.3. Normalization, Comparability, and Sensitivity

The numerical values of $\Phi_{\mathrm{PGM}}$ are comparable only within a declared signal class, control volume, timescale, normalization scheme, weight vector, and data-quality model. Otherwise, they should be interpreted as target-specific screening descriptors. Reference scales such as $E_{\mathrm{ref}}$, $A_{\mathrm{ref}}$, $\lambda_{\mathrm{ref}}$, and $\sigma_{\mathrm{cyc}}$ must be fixed before scoring and should be varied in sensitivity analysis. Applications using different reference scales should not be ranked unless a common normalization or an explicit hierarchical calibration has been supplied. For example, $E_{\mathrm{ref}}$ could be defined as the redox work required to sustain a declared reaction family over the modeled residence time; $A_{\mathrm{ref}}$ could be the reactive, fluid-accessible mineral surface area in the chosen control volume rather than total mineral abundance; and $\lambda_{\mathrm{ref}}$ should be tied to the same graph construction, feasibility filters, and edge weights used to compute $\lambda_{2}$. These examples are reporting conventions, not universal constants.

$\Phi_{\mathrm{PGM}}$ is therefore a decision-analytic screening functional, not a thermodynamic state function or universal physical observable. The regularization constant $\varepsilon_{\Phi }$ is a numerical device used to avoid logarithmic singularities; it is not physical support for a component that is absent. If a required component is below detection, physically inaccessible, or undefined for the declared signal class, the unregularized limiting behavior and the limiting component must be reported. Until calibrated against independent low-organization abiotic, prebiotic-geochemical, and biological controls, $\Phi_{\mathrm{PGM}}$ should be used as a component-wise screening descriptor rather than as a thresholded decision metric.

### 6.4. Worked Operational Example

A minimal worked example illustrates the reporting logic without claiming an empirical assessment of any specific sample. Consider a hypothetical clay-bearing lacustrine mudstone in a Noachian Mars analogue setting. The control volume is a centimetre- to metre-scale sedimentary unit, the signal class is an organic–redox–mineral association, and the timescale is the duration over which low-temperature aqueous alteration and subsequent preservation are modeled. Suppose independent measurements or models constrain the five component intervals as in Table 10. Uniform sampling within these intervals, equal five-term weights, and $\varepsilon_{\Phi }=10^{-6}$ yield a median $\Phi_{\mathrm{PGM}}\approx 0.514$ with a 5–95% interval of 0.450–0.582. The corresponding four-term organization score gives median $\Phi_{\mathrm{org}}\approx 0.464$ with a 5–95% interval of 0.394–0.541. The limiting component is $\Omega_{\mathrm{net}}$, not preservation. The intervals are didactic model inputs, not posterior estimates from Mars data.

This example shows why a single number is insufficient. The same median $\Phi_{\mathrm{PGM}}$ could arise from high organization and poor preservation, or from moderate organization and excellent preservation. Reporting $\Phi_{\mathrm{org}}$, $\Phi_{\mathrm{PGM}}$, the full component intervals, and the limiting component prevents that ambiguity.

A paired negative control is equally important. A low-organization alteration-only scenario with weak redox flux, low reactive mineral efficacy, no feasible network closure, weak recurrence, and moderate preservation gives a synthetic median $\Phi_{\mathrm{org}}=0.171$ and median $\Phi_{\mathrm{PGM}}=0.231$ in the accompanying comma-separated values (CSV) summary. This control is not a planetary estimate; it demonstrates that the score can remain low even when preservation is nonzero. It also provides a template for future calibration against sterilized analog experiments, contamination controls, and low-flux abiotic alteration sequences.

## 7. Results: Synthetic Demonstration of Model Behavior

### 7.1. Purpose and Design of the Synthetic Experiment

A theoretical index is scientifically useful only if its mathematical behavior is explicit. The following results are mathematical and synthetic; they are not empirical planetary rankings. A reproducible parameter-space demonstration is included to show how Equation (21) behaves when the five PGM components vary independently over a broad domain. The demonstration uses $M_{\mathrm{sim}}=5000$ synthetic environments generated by a Latin-hypercube design in ${\left[0.02,0.98\right]}^{5}$, with equal component weights $w_{q}=1/5$ and $\varepsilon_{\Phi }=10^{-6}$ [62]. Each synthetic environment is represented by

(27) $$x^{\left(s\right)}=\left(\Theta_{\mathrm{red}}^{\left(s\right)},\Lambda_{\mathrm{cat}}^{\left(s\right)},\Omega_{\mathrm{net}}^{\left(s\right)},\Psi_{\mathrm{cyc}}^{\left(s\right)},\Xi_{\mathrm{pre}}^{\left(s\right)}\right),\;s=1,\ldots ,M_{\mathrm{sim}}.$$

The lower and upper bounds avoid logarithmic singularities and emphasize that the exercise is a structural sensitivity demonstration, not a data assimilation procedure. In a mission or laboratory application, the uniform synthetic design would be replaced by posterior distributions obtained from thermochemical modeling, mineralogical measurements, kinetic experiments, reaction-transport calculations, or atmospheric retrievals [14,34,41,52,63].

The script, synthetic input and output tables, weak-link and negative-control summaries, and figure files used in this demonstration are supplied as Supplementary Materials; the persistent repository record is identified in the Data Availability Statement.

The geometric structure of Equation (21) has a simple local sensitivity. Differentiating Equation (23) gives

(28) $$\frac{\partial \ln\Phi_{\mathrm{PGM}}}{\partial \ln X_{q}}=\frac{w_{q}\left(1-\varepsilon_{\Phi }\right)X_{q}}{\varepsilon_{\Phi }+\left(1-\varepsilon_{\Phi }\right)X_{q}},$$

and therefore, for $X_{q}\gg \varepsilon_{\Phi }$,

(29) $$\frac{\partial \ln\Phi_{\mathrm{PGM}}}{\partial \ln X_{q}}\approx w_{q},\;\frac{\partial \Phi_{\mathrm{PGM}}}{\partial X_{q}}\approx \frac{w_{q}\Phi_{\mathrm{PGM}}}{X_{q}}.$$

Equation (29) formalizes the intended noncompensability of the index. When one component is small, the marginal gain is concentrated on that weak component under the geometric form; conversely, a high value of one component cannot fully compensate for the near absence of another. This is the mathematical reason why an energy-rich ocean world with weak archive access and an archive-rich Martian mudstone with uncertain sustained flux are not equivalent targets, even if both are astrobiologically compelling [11,12,14,48,49].

### 7.2. Projection into Organization and Archive Coordinates

**Figure 1:**
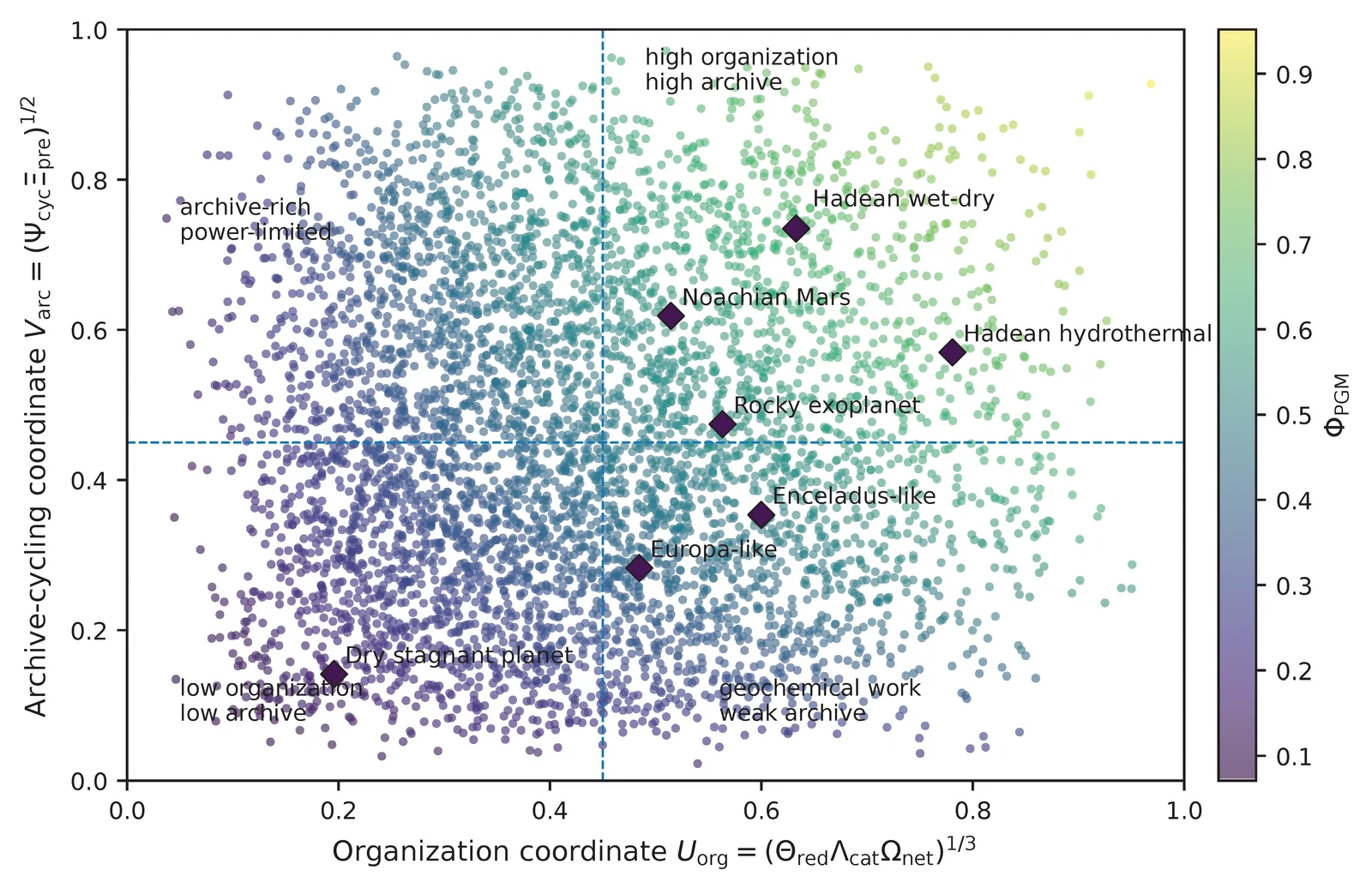
Synthetic parameter-space projection of the Geochemical Metabolic Potential. Each small point is one of $M_{\mathrm{sim}}=5000$ nondimensional synthetic environments sampled over the five PGM components. Color indicates $\Phi_{\mathrm{PGM}}$. Large diamond markers are synthetic heuristic anchors listed in Table 11; their positions are not empirical estimates, mission priorities, or cross-body rankings. The figure shows that high geochemical organization and high preservation-cycling support are separable: an environment can be energetic and catalytic but weakly archived, or strongly archived but less clearly powered. The labels designate conceptual regimes rather than fixed decision boundaries or mission priorities.

For visualization, the five-dimensional PGM space is projected onto two diagnostic coordinates:

(30) $$U_{\mathrm{org}}={\left(\Theta_{\mathrm{red}}\Lambda_{\mathrm{cat}}\Omega_{\mathrm{net}}\right)}^{1/3},$$

which measures the overlap of redox work, catalytic interfaces, and network closure, and

(31) $$V_{\mathrm{arc}}={\left(\Psi_{\mathrm{cyc}}\Xi_{\mathrm{pre}}\right)}^{1/2},$$

which measures the overlap of cycling and preservation. The notation is intentionally separate from the thermodynamic control volume V and from Gibbs free energy. The dashed lines in Figure 1 are illustrative visual guides at $U_{\mathrm{org}}=0.45$ and $V_{\mathrm{arc}}=0.45$, not universal thresholds. The labelled anchor points are pedagogical stress tests of the index and should not be read as empirical scores for real bodies, mission priorities, or cross-body rankings.

**Table 11:** Illustrative anchor scores used only for the numerical demonstration in Figure 1. Values are nondimensional heuristic ordinal inputs used to probe model behavior at declared local scales; they are not empirical measurements, calibrated priors, universal rankings, global planetary estimates, or priority estimates. Differences among illustrative environments should not be interpreted at a resolution finer than the assumed uncertainty of the ordinal inputs.

Anchor	$\Theta_{\mathrm{red}}$	$\Lambda_{\mathrm{cat}}$	$\Omega_{\mathrm{net}}$	$\Psi_{\mathrm{cyc}}$	$\Xi_{\mathrm{pre}}$	$\Phi_{\mathrm{PGM}}$	$U_{\mathrm{org}}$	$V_{\mathrm{arc}}$	Interpretation
Hadean hydrothermal	0.85	0.80	0.70	0.65	0.50	0.688	0.781	0.570	Strong work-interface-network overlap; preservation less certain.
Hadean wet–dry	0.60	0.65	0.65	0.90	0.60	0.672	0.633	0.735	Strong cycling and concentration with moderate redox work.
Noachian Mars	0.55	0.55	0.45	0.45	0.85	0.554	0.514	0.618	Archive-favorable but dependent on evidence for sustained flux.
Enceladus-like	0.80	0.60	0.45	0.50	0.25	0.486	0.600	0.354	Energetically promising, but archive and source-history constraints dominate.
Europa-like	0.65	0.50	0.35	0.40	0.20	0.391	0.485	0.283	Possible geochemical work with weak accessible preservation.
Rocky exoplanet	0.65	0.55	0.50	0.50	0.45	0.526	0.563	0.474	Moderate inferred organization, strongly marginalized over unknown geology.
Dry stagnant planet	0.25	0.20	0.15	0.10	0.20	0.172	0.196	0.141	Low organization and low preservation-cycling support.

The numerical demonstration yields three methodological consequences. First, $\Phi_{\mathrm{PGM}}$ is not a proxy for habitability alone. Enceladus-like and Europa-like cases may retain substantial $\Theta_{\mathrm{red}}$ because water–rock reactions can supply chemical energy, yet their projected $V_{\mathrm{arc}}$ remains low if accessible preservation and source-region continuity are poorly constrained [14,48,49,50]. Second, archive-rich settings do not automatically become high-PGM environments. A Noachian Mars archive is compelling precisely because $\Xi_{\mathrm{pre}}$ can be high, but its interpretation still depends on redox–mineral–organic coupling, alteration history, and evidence for repeated geochemical flux [11,12,13,46,61]. Third, the model gives a practical reporting language: more informative proto-biosignature hypotheses are not merely associated with the wettest environments, but with declared implementations in which $U_{\mathrm{org}}$, $V_{\mathrm{arc}}$, $X_{\lim}$, and $\Phi_{\mathrm{PGM}}$ are reported together.

The synthetic anchors should not be read as empirical rankings. They are boundary tests for the mathematics. Their role is analogous to a sensitivity experiment: they show how the model reacts when energy, catalysis, network closure, cycling, and preservation are varied independently. The weak-link audit in Table 12 is therefore as important as the scalar value itself. A real application would replace these ordinal inputs with posterior component distributions, and its most important output would often be the uncertainty structure rather than the median value of $\Phi_{\mathrm{PGM}}$.

Two additional stress tests are included in the supplementary CSV outputs. In a one-low-component case with X=(0.90,0.90,0.02,0.90,0.90), the scalar value remains $\Phi_{\mathrm{PGM}}\approx 0.420$ because the geometric mean is regularized and finite, but the limiting component is $\Omega_{\mathrm{net}}=0.02$. This is why $\Phi_{\mathrm{PGM}}$ must never be reported without $\min_{q}X_{q}$ and the full component vector. A second feasibility-mask check retains only synthetic environments satisfying $\Omega_{\mathrm{net}}\leq {\left(\Theta_{\mathrm{red}}\Lambda_{\mathrm{cat}}\right)}^{1/2}$; the retained fraction is 0.447 and the median $\Phi_{\mathrm{PGM}}$ remains 0.412. This check does not validate any planetary distribution, but it shows that the qualitative behavior of the index is not an artifact of allowing arbitrarily high network closure when redox work and catalytic support are weak.

## 8. Proto-Biosignature Classes

Table 13 summarizes major proto-biosignature classes. The table emphasizes that each class is ambiguous alone and gains meaning only through context. This is the conceptual difference between a proto-biosignature and a diluted biosignature: ambiguity is not hidden, but made explicit and used to structure further tests [27,37,38,39,42].

### 8.1. Proto-Biosignatures Versus Potential Biosignatures

A potential biosignature is a candidate signal that may ultimately support a biological interpretation after false positives, false negatives, context, and measurement reliability are assessed. A proto-biosignature has a narrower and lower evidential status. It supports $H_{\mathrm{pg}}$–prebiotic geochemical organization–more directly than it supports $H_{\mathrm{bio}}$. This distinction makes proto-biosignatures complementary to biosignature-threshold reasoning and LDKB-style taxonomies: they help decide whether a sample or planet occupied a chemically organized prebiotic regime before any biological attribution is attempted [27,36,37,38].

### 8.2. Redox-Mineral-Organic Associations

A particularly important proto-biosignature class is not an isolated molecule but a contextual association: organics, redox-sensitive minerals, catalytic phases, and depositional or hydrothermal setting. On Mars, fine-grained sediments and redox-sensitive mineral assemblages are attractive because they can preserve organics and record chemical gradients; on Enceladus, hydrogen, salts, phosphates, and organic compounds point to ongoing ocean chemistry; on early Earth, hydrothermal and sedimentary archives show how mineralogical context can organize and preserve signals [11,12,47,48,49,50]. The PGM model predicts that more informative proto-biosignature cases will involve co-localization rather than co-occurrence: the relevant phases should be spatially, stratigraphically, or temporally coupled.

Such associations are also the most vulnerable to false positives. Redox fronts can form abiotically; organics can be delivered by meteorites or synthesized by geochemical processes; iron and sulfur minerals can form during diagenesis; and textural patterns may arise from physical or chemical self-organization. For that reason, the model requires explicit comparison between $H_{\mathrm{ab}}$ and $H_{\mathrm{pg}}$ rather than direct inference to biology [1,27,39,42]. A proto-biosignature is valuable precisely because it identifies where further tests should be concentrated.

### 8.3. Molecular Complexity and Agnostic Features

Molecular-complexity metrics have become prominent because they aim to detect the effects of selection, history, or constrained assembly without assuming terrestrial biochemistry. Assembly theory and machine-learning approaches to mass spectra represent two active lines of agnostic biosignature research [28,29,30,31]. In the present model, such metrics can act as proto-biosignatures when high-complexity signals occur in a geochemical context compatible with production, concentration, and preservation. Without context, complexity is underdetermined: abiotic chemistry can produce complex mixtures, and analytical workflows can transform or bias molecular distributions.

The PGM interpretation is therefore conditional. A high molecular assembly index, a complex mass spectral pattern, or a non-random molecular distribution increases relevance when it is tied to $\Theta_{\mathrm{red}}$, $\Lambda_{\mathrm{cat}}$, $\Omega_{\mathrm{net}}$, and $\Xi_{\mathrm{pre}}$. A complex molecule in a sterile contamination pathway is not a proto-biosignature. A simpler but spatially coherent organic–mineral–redox pattern may be more informative. This view aligns agnostic biosignature research with geological context rather than placing molecular complexity outside geochemistry [28,29,60,64].

### 8.4. Minerals as Catalytic and Archival Information

Mineral evolution provides a powerful Earth-science analogue for thinking about non-biological information. Minerals diversify through planetary differentiation, fluid–rock interaction, atmospheric oxidation, biological activity, and historical contingency; their occurrence patterns and paragenetic modes encode processes that are not reducible to bulk composition [32,33,63,64]. Within PGM, minerals have two roles. They participate in reaction networks as catalysts, adsorbents, compartments, and electron-transfer media; and they preserve an archive of chemical environments.

This dual role is central to proto-biosignatures. Transition-metal sulfides, native metals and alloys, magnetite, mixed-valence oxides, clays, phosphates, carbonates, silica, sulfates, and redox-sensitive oxides are not proto-biosignatures simply by existing. They become proto-biosignature components when their paragenesis, texture, redox state, and organic associations indicate persistent chemical flux through a system capable of prebiotic organization [11,12,17,21,55,57]. Mineralogical context thus provides the archive in which geochemical metabolism can become observable.

## 9. Bayesian Interpretation and the Status of Proto-Biosignatures

### 9.1. Three Hypotheses Rather than Two

Life-detection discussions often compare a biological hypothesis with an abiotic false-positive hypothesis. Proto-biosignatures require at least three generative hypotheses for the observed feature vector:

$$\begin{array}{cc} H_{\mathrm{ab}} & :\mathrm{low}\text{-}\mathrm{organization}\;\mathrm{abiotic}\;\mathrm{chemistry},\\ H_{\mathrm{pg}} & :\mathrm{non}\text{-}\mathrm{biological}\;\mathrm{prebiotic}\;\mathrm{geochemical}\;\mathrm{organization},\\ H_{\mathrm{bio}} & :\mathrm{extant}\;\mathrm{or}\;\mathrm{extinct}\;\mathrm{biology}. \end{array}$$

Here, $H_{\mathrm{pg}}$ means that prebiotic geochemical organization, not biological activity, generates the candidate feature vector. Given data d and contextual information $C_{\mathrm{env}}$, the posterior probability of hypothesis $H_{k}$ is

(32) $$P\left(H_{k}|d,C_{\mathrm{env}}\right)=\frac{P\left(d|H_{k},C_{\mathrm{env}}\right)P\left(H_{k}|C_{\mathrm{env}}\right)}{\sum_{\ell }P\left(d|H_{\ell },C_{\mathrm{env}}\right)P\left(H_{\ell }|C_{\mathrm{env}}\right)}.$$

A candidate feature vector b is a proto-biosignature candidate when it raises the Bayes factor

(33) $$K_{\mathrm{pg}/\mathrm{ab}}=\frac{P(b|H_{\mathrm{pg}},C_{\mathrm{env}})}{P(b|H_{\mathrm{ab}},C_{\mathrm{env}})}$$

above unity. This condition indicates directional support only. Operational use should define evidence categories before feature evaluation, for example, weak support when $K_{\mathrm{pg}/\mathrm{ab}}>1$ but the uncertainty interval includes negligible support, moderate support when a preregistered threshold is exceeded with uncertainty accounted for, and strong support only when an independently declared threshold and negative controls are satisfied. The criterion is therefore a minimal candidate criterion, not a claim of strong proto-biosignature evidence. A candidate feature vector may satisfy this condition without necessarily raising

(34) $$K_{\mathrm{bio}/\mathrm{ab}}=\frac{P(b|H_{\mathrm{bio}},C_{\mathrm{env}})}{P(b|H_{\mathrm{ab}},C_{\mathrm{env}})}$$

to a level sufficient for a life claim. This three-hypothesis structure is consistent with calls for probabilistic, contextual, and staged life-detection reasoning [3,4,5,6,27,39].

The same relation can be written as posterior odds between prebiotic geochemical organization and the abiotic null:

(35) $$\frac{P(H_{\mathrm{pg}}|d,C_{\mathrm{env}})}{P(H_{\mathrm{ab}}|d,C_{\mathrm{env}})}=K_{\mathrm{pg}/\mathrm{ab}}\;\frac{P(H_{\mathrm{pg}}|C_{\mathrm{env}})}{P(H_{\mathrm{ab}}|C_{\mathrm{env}})}.$$

This expression clarifies the evidential status of proto-biosignatures. The feature vector b contributes through the likelihood ratio, whereas $\Phi_{\mathrm{PGM}}$ contributes through environmental context or through explicitly modeled nuisance parameters. The same observation must not be used twice, once to construct the context score and again to claim an independent likelihood gain.

### 9.2. Connection Between $\Phi_{\mathrm{PGM}}$ and Evidence

The organization score and the detectable-evidence score have distinct Bayesian roles. For origin-focused inference, $\Phi_{\mathrm{org}}$ is the cleaner context prior for prebiotic geochemical organization because it excludes the archive term. $\Xi_{\mathrm{pre}}$ and $\Phi_{\mathrm{PGM}}$ are better treated as recoverability or observation factors unless the inferential target is explicitly the probability of finding a preserved proto-biosignature. Thus, in a three-hypothesis analysis, the safest use is as prior odds between prebiotic geochemical organization and the low-organization abiotic null:

(36) $$\frac{P(H_{\mathrm{pg}}|C_{\mathrm{env}})}{P(H_{\mathrm{ab}}|C_{\mathrm{env}})}=\frac{\Phi_{\mathrm{org}}^{\eta }}{\beta_{\mathrm{ab}}},$$

where η>0 controls sensitivity to the organization score and $\beta_{\mathrm{ab}}>0$ sets the baseline odds assigned to low-organization abiotic chemistry. Equation (36) is illustrative; η and $\beta_{\mathrm{ab}}$ must be fixed before examining candidate feature vectors or learned from independent controls. Its purpose is to show how PGM can be connected to formal inference without equating high geochemical potential or high preservation potential with life.

A normalized three-hypothesis prior requires explicit weights for all hypotheses. One transparent construction is

(37) $$\omega_{\mathrm{ab}}=1,\;\omega_{\mathrm{pg}}=\frac{\Phi_{\mathrm{org}}^{\eta }}{\beta_{\mathrm{ab}}},\;\omega_{\mathrm{bio}}=\gamma_{\mathrm{bio}},$$

with $\gamma_{\mathrm{bio}}$ specified independently from prior biological plausibility, target history, or external controls. If the inferential target is recoverable evidence rather than organization itself, the observation model may include $\Xi_{\mathrm{pre}}$ or $\Phi_{\mathrm{PGM}}$ explicitly, but that choice must be stated before likelihood evaluation. The normalized prior is then

(38) $$P\left(H_{k}|C_{\mathrm{env}}\right)=\frac{\omega_{k}}{\omega_{\mathrm{ab}}+\omega_{\mathrm{pg}}+\omega_{\mathrm{bio}}},\;k\in \left\{\mathrm{ab},\mathrm{pg},\mathrm{bio}\right\}.$$

This construction makes clear that a high $\Phi_{\mathrm{org}}$ increases the plausibility of $H_{\mathrm{pg}}$ relative to $H_{\mathrm{ab}}$, not necessarily $H_{\mathrm{bio}}$ [6,27,39,42]. Direct use of $\Phi_{\mathrm{PGM}}$ in prior odds is defensible only when the observational role of preservation has been explicitly justified.

Data partitioning is required to avoid circular inference. The partition between contextual data $d_{X}$ and candidate features b should be declared before evaluating a candidate signal; otherwise the dependence must be represented through an explicit joint likelihood. Variables used to construct the component vector X are contextual covariates. Candidate features used in $K_{\mathrm{pg}/\mathrm{ab}}$ must be conditionally independent of those covariates after the stated context is fixed, or they must be modeled through an explicit joint likelihood,

(39) $$P\left(b,X|H_{k},C_{\mathrm{env}}\right)=P\left(b|X,H_{k},C_{\mathrm{env}}\right)P\left(X|H_{k},C_{\mathrm{env}}\right).$$

A redox–organic association, for example, cannot both define the context score and independently raise the likelihood ratio unless the dependence is represented in Equation (39).

For the mudstone example above, one admissible partition would use bulk mineralogy, redox-state constraints, alteration temperature, and host preservation to estimate $d_{X}\to X$. The feature vector b would then retain only conditionally separate observations, such as spatial heterogeneity of organic molecular distributions, isotope-pattern coherence at a scale not used in X, or independent blank-corrected mass-spectral structure. If redox-sensitive mineral assemblages and organics are co-localized and that co-localization is already used in $\Xi_{\mathrm{pre}}$ or $\Theta_{\mathrm{red}}$, it must enter the likelihood through Equation (39), not as an independent Bayes-factor multiplier. Table 14 presents the reporting logic.

The model also helps structure part of the problem of unconceived alternatives. Unknown abiotic processes cannot be eliminated by definition, but their impact can be reduced by requiring that multiple independent terms of $\Phi_{\mathrm{PGM}}$ support the same interpretation. A signal that requires redox disequilibrium, mineral catalysis, network connectivity, environmental cycling, and preservation to align is less vulnerable than a signal based on one molecule or one texture. This is not evidence sufficient for life; it is a disciplined way to prioritize hypothesis testing and additional measurements [3,37,38,39].

## 10. Discussion: Planetary Applications

### 10.1. Hadean-Eoarchean Earth

Early Earth supplies the reference problem because it is the only planet known to have crossed from geochemistry into biology. The PGM model does not choose a single origin-of-life setting. Instead, it evaluates why several proposed settings are plausible in different terms. Alkaline hydrothermal vents score highly in redox exergy, mineral compartmentalization, pH gradients, and transition-metal sulfide, native-metal, alloy, and mixed-valence-surface catalysis. Wet–dry surface environments score highly in environmental cycling, concentration, and condensation chemistry. Impact, volcanic, evaporitic, and sedimentary settings may contribute feedstocks and preservation under different conditions [9,17,18,20,23,24,45].

A proto-biosignature approach suggests that the origin of life should not be reconstructed from biomolecule synthesis alone. It should be reconstructed from the co-occurrence of energy flux, catalytic mineral surfaces, network closure, cycling, and preservation. This perspective can reconcile part of the hydrothermal-surface debate: different environments may supply different PGM components, and life’s emergence may have required exchange between them. Hydrothermal systems may provide sustained redox energy; surface cycling may provide concentration and polymerization; sediments and minerals may provide preservation and selection of molecular ensembles [19,21,22,24,57].

### 10.2. Noachian Mars

Mars is the archetype of an archival world. Ancient Mars possessed fluvio-lacustrine environments, clay minerals, sulfate deposits, redox gradients, igneous–water interactions, and sedimentary settings capable of preserving organic and mineralogical signals [11,12,13,46,61]. In PGM terms, Mars may have had spatially heterogeneous values of $\Phi_{\mathrm{PGM}}$: high in hydrothermal, lacustrine, deltaic, and groundwater-influenced environments, lower in oxidized or strongly radiation-exposed surfaces, and strongly dependent on burial and diagenetic history.

The important consequence is that Mars targets should be evaluated not only by past habitability but by overlap among PGM terms. A clay-rich lacustrine mudstone with redox-front mineral assemblages, organic preservation, and stratigraphic evidence for sustained water–rock interaction is a more informative proto-biosignature context than a generic hydrated deposit. Recent redox–mineral–organic associations reported in Jezero Crater illustrate why such contexts are scientifically powerful yet still require caution: they may be consistent with processes relevant to microbial metabolism on Earth, but abiotic diagenetic and redox pathways must be evaluated before any biological inference [27,39,46,47,61,65]. A Mars-specific reactor–filter treatment of partially decoupled synthesis, metabolic plausibility, and preservation windows provides a complementary target-specific example of this logic [16]. In this interpretation, the conservative conclusion is not “life”, but elevated $K_{\mathrm{pg}/\mathrm{ab}}$ and a strong case for sample return and multi-instrument analysis. Nickel-rich associations co-located with redox–organic interactions in Neretva Vallis provide another example of why metal enrichment, organics, and redox context should be interpreted as proto-biosignature-relevant context rather than as stand-alone evidence for biogenicity [65].

### 10.3. Europa, Enceladus, and Ocean Worlds

Ocean worlds invert the Martian problem. Enceladus and Europa may host active water–rock systems, salts, redox gradients, and chemical exchange between ocean, rock, ice, and space. Enceladus provides particularly direct evidence of ocean materials through plume sampling: molecular hydrogen, sodium phosphates, and complex organic compounds support the interpretation of ongoing geochemical activity and habitability-relevant chemistry [48,49,50,66]. Europa, meanwhile, is a major target because its ocean, ice shell, surface exchange, and geological activity may allow assessment of habitability by Europa Clipper and future missions [14,15].

In PGM terms, ocean worlds may score highly in $\Theta_{\mathrm{red}}$ and possibly $\Lambda_{\mathrm{cat}}$ if ocean–rock interaction is sustained. They may score variably in $\Omega_{\mathrm{net}}$ depending on residence times, feedstocks, and destruction pathways. Their most challenging term is often $\Xi_{\mathrm{pre}}$: active chemistry may be present, but the archive may be hidden beneath ice or altered during transport and plume ejection. Proto-biosignatures in plume material should therefore be interpreted as samples of ongoing water–rock geochemical activity, not necessarily preserved histories [14,48,49,50]. A null biological result in such a high-PGM environment would itself be theoretically informative, because it would constrain which combinations of energy, chemistry, and time are insufficient under the sampled conditions.

### 10.4. Terrestrial Exoplanets

For exoplanets, direct mineralogical and sedimentological context is usually unavailable, so PGM must be inferred indirectly as a posterior predictive distribution rather than as a point score. Atmospheric chemical disequilibrium, especially combinations such as O_2_ or O_3_ with reduced gases, has been proposed as a metabolism-agnostic biosignature, but recent work shows that retrieval uncertainty, photochemistry, stellar context, and false positives complicate interpretation [2,34,35,41]. The PGM model does not replace atmospheric biosignature assessment; it adds a geological prior. A disequilibrium atmosphere is more informative when the planet plausibly maintains surface-interior exchange, volatile cycling, redox gradients, and long-term environmental stability.

This point is especially important for putative biosignature gases. A gas cannot be interpreted without source, sink, context, and competing abiotic production pathways. The PGM approach asks whether the inferred atmosphere is compatible with planetary processes that could sustain geochemical or biological disequilibrium, and whether alternative geological and photochemical models have been marginalized. For exoplanets, proto-biosignatures may therefore be probabilistic patterns in atmospheric composition and temporal variability that raise $K_{\mathrm{pg}/\mathrm{ab}}$ or $K_{\mathrm{bio}/\mathrm{ab}}$ only after marginalization over stellar context, volatile cycling, interior redox state, surface exchange, and competing photochemical or geochemical false positives [1,2,34,35,41].

Table 15 summarizes these qualitative contrasts across planetary regimes.

Two practical implications follow from this comparison. First, the most informative target is not necessarily the one with the largest inventory of any single ingredient. It is the target in which independent observables constrain the same process history: energy flow, mineral mediation, network coupling, recurrence, and preservation. Second, the model separates scientific priority from evidential strength. Enceladus may be a high-priority target because active geochemical work is accessible, whereas Mars may be a high-priority target because ancient archives are recoverable. In neither case does priority imply biogenicity; it identifies where additional measurements can most efficiently reduce uncertainty among the three hypotheses.

## 11. Calibration, Uncertainty, and Domain of Validity

The numerical values used in Table 11 are illustrative ordinal inputs, not measured planetary scores. This distinction is important. The model becomes quantitative for a specific target only after the five component terms are calibrated against an explicit control volume, timescale, signal class, and measurement model. A clay-bearing Martian mudstone, a hydrothermal mound, an Enceladus ice grain, and a terrestrial exoplanet atmosphere are not commensurate samples until their observables have been mapped into the same nondimensional component definitions. For this reason, $\Phi_{\mathrm{PGM}}$ should be reported together with its assumptions, weights, uncertainty ranges, regularization constant, $X_{\lim}$, and weak-link flag, rather than as a single context-free number. If $X_{\lim}$ falls below a declared $X_{\mathrm{thr}}$, the target should be described as weak-link constrained even when the scalar $\Phi_{\mathrm{PGM}}$ appears moderate.

Uncertainty enters the model at several levels. Thermodynamic databases and activities affect $\Theta_{\mathrm{red}}$; reactive surface area and rate constants affect $\Lambda_{\mathrm{cat}}$; incomplete reaction inventories affect $\Omega_{\mathrm{net}}$; residence times and recurrence histories affect $\Psi_{\mathrm{cyc}}$; and degradation, diagenesis, radiation, and contamination affect $\Xi_{\mathrm{pre}}$. In a mature implementation, each component should therefore be treated as a probability distribution rather than as a point estimate. Equation (21) then becomes a propagated distribution for $\Phi_{\mathrm{PGM}}$, and the relevant output is not only the median score but also the probability that the target lies in a proto-biosignature-favorable region of parameter space [34,37,38,39,52].

The domain of validity is also bounded. PGM is designed for environments in which water–rock–fluid reactions, including atmospheric, ice-shell, or gas-phase boundary conditions when relevant, mineral interfaces, and preservation pathways can be specified at least approximately. It is less directly applicable to highly reducing gas giants, purely atmospheric photochemical systems without recoverable surface or interior context, or samples whose contamination history dominates the signal. It also does not solve the origin of heredity, translation, or cellular individuality. Instead, it addresses an earlier physical question: whether a planetary environment can maintain the energy flow, catalytic coupling, network structure, recurrence, and archive quality required for prebiotic organization to become detectable. This limited scope is what makes the model testable.

Two safeguards are especially important. The first is independence and data partitioning. If a redox–organic association is used to estimate $\Theta_{\mathrm{red}}$ or $\Lambda_{\mathrm{cat}}$, it should not then be treated as an independent feature supporting the same hypothesis unless the likelihood model accounts for that dependence. The second is negative calibration. Low-$\Phi_{\mathrm{PGM}}$ control environments, sterilized analog experiments, and purely abiotic alteration sequences are as important as positive cases because they determine how often proto-biosignature-like patterns arise without prebiotic organization. Without such controls, the theory would risk becoming descriptive rather than discriminating.

Table 16 summarizes the minimum benchmarking ladder needed before target-specific use. These controls define the null distribution of proto-biosignature-like patterns and the conditions under which the model should fail.

## 12. Predictions and Falsifiability

The model makes five falsifiable predictions. They are phrased as tests because the value of the model depends on its ability to fail under contrary evidence.

*Prediction 1: overlap should outperform inventory.* Proto-biosignature richness should correlate more strongly with the spatial overlap of redox gradients, catalytic mineral phases, reaction products, and preservation-favorable hosts than with water indicators alone. On Mars, this predicts that fine-grained, redox-stratified, clay- or carbonate-bearing sedimentary rocks should be more informative than hydrated rocks lacking organic–mineral–redox coupling [11,12,46,47]. On early Earth analogues, it predicts that geochemical interfaces with repeated flux and catalytic mineral diversity should produce more structured organic distributions than equilibrium aqueous mixtures [21,24,57,59].

*Prediction 2: active chemistry and recoverable evidence can decouple.* A high-$\Theta_{\mathrm{red}}$ ocean-world seafloor with low $\Xi_{\mathrm{pre}}$ may support extensive geochemical organization while leaving few accessible archives. Conversely, an ancient Martian sediment may have high $\Xi_{\mathrm{pre}}$ but uncertain current or past $\Theta_{\mathrm{red}}$. Mission strategies optimized for habitability and those optimized for evidence recovery should therefore diverge in some targets [11,14,48,49,50].

*Prediction 3: context should dominate single-marker interpretation.* A single molecule, isotope ratio, color, spectral edge, or mineral texture should rarely dominate interpretation. Strong proto-biosignature candidates should involve cross-consistency among thermodynamic models, mineral paragenesis, molecular distributions, isotopic constraints, and preservation models. This agrees with modern biosignature assessment, but adds $H_{\mathrm{pg}}$ as a necessary intermediate hypothesis between abiotic null and biology [3,5,27,37,38,39].

*Prediction 4: molecular complexity should be conditional on PGM context.* A complex organic mixture detected in a high-energy, catalytic, cycling, preservation-favorable environment should raise $K_{\mathrm{pg}/\mathrm{ab}}$ more than the same mixture detected without geological context. This prediction can be tested experimentally by comparing molecular-complexity metrics across controlled abiotic, prebiotic, and biological samples under known mineral and cycling conditions [27,28,29,30,31].

*Prediction 5: failure cases should revise the model.* If high-$\Phi_{\mathrm{PGM}}$ environments repeatedly fail to produce network closure, molecular complexity, or proto-biosignature patterns in laboratory simulations and planetary samples, then the assumed link between PGM-like geochemical organization and abiogenesis must be weakened. Conversely, if low-$\Phi_{\mathrm{PGM}}$ environments frequently produce strong proto-biosignature patterns, the weighting, component definitions, or underlying model must be revised. The value of $\Phi_{\mathrm{PGM}}$ lies in making those failures interpretable rather than anecdotal.

The model is therefore empirically vulnerable in a useful sense. It would be weakened by controlled experiments showing that redox exergy, catalytic mineral surfaces, network closure, cycling, and preservation do not increase the frequency or coherence of prebiotic feature vectors relative to abiotic controls. It would also be weakened by planetary samples in which predicted high-overlap environments consistently lack proto-biosignature classes while low-overlap environments consistently exhibit them. These are demanding tests, but they are precisely what make the proposal more than a terminological addition.

## 13. Experimental and Technical Requirements for PGM Implementation

The model is intentionally written so that its present limitations are visible. A useful PGM calculation requires measurements that are rarely obtained together: redox speciation, mineral surface reactivity, reaction-network feasibility, recurrence or residence time, and preservation state at the same declared scale. Table 17 translates each PGM component into observables, available techniques, current limitations, and development needs. The table is not a claim that all measurements are currently available for all targets. On the contrary, identifying missing observables is part of the value of the model, because it shows which measurement would most reduce uncertainty.

Several consequences follow. First, PGM cannot be implemented by measuring organics alone. It requires co-located geological, mineralogical, chemical, and temporal information. Second, the most valuable future measurements are not necessarily more sensitive detections of one compound, but measurements that link a candidate compound to redox state, mineral host, catalytic surface, fluid history, and preservation pathway. Third, where methods are lacking, the missing component should be reported explicitly rather than filled with a terrestrial default. A high-quality application may therefore conclude that $\Phi_{\mathrm{PGM}}$ is presently underdetermined. Such a conclusion is not a failure of the model; it is a useful statement about what must be measured next.

## 14. Research Program

A research program built on this model would require three linked efforts. The first is experimental and should be factorial rather than merely descriptive. Analog systems should vary redox exergy, mineral catalytic surfaces, cycling amplitude, and preservation host independently, while holding contamination and analytical history under control. A minimal factorial design would compare high/low redox drive, catalyst present/absent, cycling present/absent, and preservation host favorable/unfavorable with low-PGM abiotic controls, alteration-only controls, and contamination controls. These low-PGM controls are needed not only for false-positive rejection, but also to estimate the null distribution of proto-biosignature-like patterns. The primary observables should include not only target biomolecules, but reaction-network structure, molecular-complexity distributions, isotope patterns, mineral paragenesis, and signal survival after alteration. Existing experiments on hydrothermal systems, wet–dry chemistry, clay catalysis, metal-promoted carbon fixation, and molecular complexity provide the necessary foundation [23,24,28,56,57,59].

The second effort is computational. Geochemical reaction-transport models, thermodynamic speciation, kinetic networks, graph-theoretic closure analysis, and Bayesian evidence models should be integrated rather than treated as separate disciplines. A PGM model for a target environment should output not only predicted mineral or gas abundances, but expected feature vectors b under $H_{\mathrm{ab}}$, $H_{\mathrm{pg}}$, and $H_{\mathrm{bio}}$. This is feasible in principle because disequilibrium modeling, atmospheric retrieval, reaction-network thermodynamics, and mineral association analysis are already mature enough to be coupled [1,34,41,52,63].

The third effort is mission-oriented. Instruments and sampling strategies should be designed to measure component overlap at the same spatial and temporal scale: redox state, mineralogy, native-metal/alloy or sulfide/oxide phases when present, organic chemistry, isotopic composition, textural context, and preservation state. The ideal measurement is not merely a stronger organic detection, but a co-registered observation in which organics, redox-sensitive mineral assemblages, native metals or alloys when present, catalytic phases, host mineralogy, and stratigraphic or plume context can be interpreted together. Mars sample return, ocean-world plume sampling, Europa Clipper, and future exoplanet observatories would all benefit from sampling and measurement criteria that distinguish habitability from geochemical organization and evidence preservation [11,12,14,15,35,37].

A practical near-term test would compare three sample classes under shared analytical protocols: low-organization abiotic controls, experimentally organized prebiotic geochemical systems, and biological or fossil-biological reference materials. The model predicts that proto-biosignature candidates should occupy an intermediate region: higher in contextual organization than abiotic controls, but lacking the full evidential structure required for biological attribution. Failure of this separation would be informative, because it would show which PGM components or weighting assumptions require revision.

## 15. Current Barriers and Development Pathways

The present state of the model should be understood as a research program rather than a finished field instrument. Table 18 summarizes the principal barriers that currently limit PGM implementation and the development pathways that would make the model increasingly useful for astrobiologists, geochemists, and origin-of-life researchers.

These barriers do not make the model unusable. They define the conditions under which it becomes scientifically useful. A target with missing redox fluxes, unresolved mineral surface area, or unknown preservation history should not be forced into a precise score. Instead, the unresolved term should be carried forward as uncertainty, a weak-link flag, or a stated measurement requirement. This is the main practical role of PGM at the present stage: to convert an ambiguous astrobiological impression into a transparent list of measurements, assumptions, and tests.

## 16. Conclusions

This paper has developed a thermodynamically grounded model linking Earth sciences and astrobiology. It is not a complete model of abiogenesis. It formalizes a narrower, testable interval: environments in which water–rock–fluid disequilibria, catalytic interfaces, network coupling, recurrence, and preservation jointly increase the plausibility of prebiotic geochemical organization relative to low-organization abiotic chemistry.

The Geochemical Metabolic Potential, $\Phi_{\mathrm{PGM}}$, formalizes this interval through five nondimensional components: $\Theta_{\mathrm{red}}$ for redox-exergy availability, $\Lambda_{\mathrm{cat}}$ for catalytic interfaces, $\Omega_{\mathrm{net}}$ for reaction-network closure, $\Psi_{\mathrm{cyc}}$ for environmental cycling, and $\Xi_{\mathrm{pre}}$ for preservation. The mathematical structure is informed by chemical affinity, Gibbs free energy, entropy production, redox thermodynamics, reaction-network connectivity, kinetic cycling, and signal survival. It is deliberately not a detector of life. Its function is to identify environments in which prebiotic geochemical organization is physically plausible, observationally recoverable, and open to falsification.

Proto-biosignatures follow from this distinction. They are neither biosignatures nor uninteresting abiotic signals. They are contextual patterns that raise the likelihood of prebiotic geochemical organization relative to low-organization abiotic chemistry. Their value lies in guiding hypothesis selection, measurement design, and context-specific sampling rationale while preserving the caution demanded by false positives and unconceived alternatives. The model is therefore conservative in evidential claims but comparatively useful across target classes: it connects early Earth, Mars, ocean worlds, and exoplanets through a common language of gradients, interfaces, networks, cycles, and archives. $\Phi_{\mathrm{PGM}}$ is a screening and hypothesis-generating quantity; empirical calibration, low-PGM controls, and independent biological or fossil-biological controls remain required for target-specific inference. No target-specific scalar $\Phi_{\mathrm{PGM}}$ should be reported without the component vector, declared weights, $\varepsilon_{\Phi }$, uncertainty ranges, $X_{\lim}$, $X_{\mathrm{thr}}$, and $f_{\mathrm{WL}}$.

The major present obstacles are not mathematical notation but implementation: Earth-centred analogues, scale-dependent redox heterogeneity, incomplete reaction inventories, uncertain catalytic efficacy, missing recurrence histories, and the difficulty of separating formation from preservation. The way forward is correspondingly practical: co-registered mineralogical, redox, organic, isotopic, textural, and temporal measurements; mineral-specific prebiotic reactor experiments; explicit control volumes and upscaling rules; negative controls; and transparent reporting of unresolved components. In this sense, the model does not solve the origin of life. It clarifies which measurements would make prebiotic geochemical organization testable.

## Figures and Tables

**Table 1:** Relationship between PGM and selected existing life-detection approaches.

Approach	Core Question	Limitation for the Prebiotic Interval	What PGM Adds
Staged life-detection scales, including CoLD	How strong is the confidence in a life-detection claim?	Intermediate geochemical organization may be treated only as weak biological evidence.	A non-biological hypothesis, $H_{\mathrm{pg}}$, for prebiotic organization before confidence in life is communicated.
Potential-biosignature catalogues and LDKB	Which signals, false positives, and false negatives should be evaluated?	Catalogues classify signals but do not by themselves score the geochemical process that generates them.	A thermodynamic and network basis for contextual weighting before a life claim.
Agnostic molecular complexity	Does a sample contain complexity suggestive of selection or history?	Complexity can be ambiguous without source, host phase, and alteration context.	Coupling between molecular patterns, mineral catalysis, redox work, and preservation.
Atmospheric disequilibrium analysis	Are remotely detected gases inconsistent with abiotic steady states?	Geological and interior constraints are often latent or weakly observed.	A geological prior linking atmospheric disequilibrium to possible water–rock reaction networks.

Abbreviations: CoLD, Confidence of Life Detection; LDKB, Life Detection Knowledge Base; PGM, Planetary Geochemical Metabolism.

**Table 2:** Scale ladder for target-specific PGM applications. Scores from different rows are not directly comparable unless an explicit upscaling or downscaling rule is declared.

Scale Class	Typical Control Volume	Useful Observables	Principal Caution
Mineral or microdomain	grains, pores, veins, reaction rims, microbial-analogue films	oxidation state, inclusion assemblages, reactive surface texture, co-localized organic films	Bulk composition can erase juxtaposed reduced and oxidized microdomains.
Pore to sample	core plug, hand specimen, returned sample aliquot	porosity, permeability, mineralogy, molecular inventory, textural relationships	Sample handling and contamination history can dominate weak signals.
Facies or alteration zone	bed, lamina package, hydrothermal halo, carbonate/sulfate/clay interval	stratigraphy, paragenesis, redox fronts, diagenetic overprint	Apparent co-occurrence may not imply simultaneous formation.
Hydrothermal field or basin	vent field, lacustrine basin, groundwater system, impact-heated zone	fluid flux, residence time, heat source, depositional setting	Local reaction cells cannot be replaced by a simple regional average.
Regional to crustal reservoir	crustal province, oceanic lithosphere, volcanic arc, mantle–crust–atmosphere exchange	reservoir composition, volatile cycling, differentiation history	Earth-derived priors become increasingly conditional and uncertain.
Remote planetary or exoplanetary	atmospheric column, plume source region, global spectral retrieval	gases, aerosols, surface/ice proxies, disequilibrium estimates	Context is underdetermined; PGM should be used as a prior for new measurements, not as a classification.

**Table 3:** Hydrothermal and water–rock settings relevant to PGM. The table prevents the term “hydrothermal” from being read as only submarine black-smoker chemistry or only alkaline vent chemistry.

Setting	Dominant Geochemical Issue	PGM Components Commonly Emphasized	Non-Equivalence Warning
Alkaline submarine hydrothermal systems	Serpentinization, pH gradients, H_2_ production, mineral membranes	$\Theta_{\mathrm{red}}$, $\Lambda_{\mathrm{cat}}$, $\Omega_{\mathrm{net}}$	Not all submarine vents are alkaline or serpentinization dominated.
High-temperature black-smoker systems	Sulfide precipitation, thermal gradients, metal transport, acidity	$\Theta_{\mathrm{red}}$, local $\Lambda_{\mathrm{cat}}$	High temperature can enhance flux but destroy or overprint fragile organics.
Subaerial volcanic–hydrothermal and arc systems	Magmatic volatiles, boiling, acid-sulfate alteration, metal mobility	$\Psi_{\mathrm{cyc}}$, local $\Theta_{\mathrm{red}}$, alteration archives	Arc-related systems are not interchangeable with oceanic vents.
Serpentinization-dominated ultramafic systems	Fe (II) oxidation, H_2_, methane, magnetite, native metals/alloys	$\Theta_{\mathrm{red}}$, $\Lambda_{\mathrm{cat}}$	Microscale redox heterogeneity can be stronger than bulk composition implies.
Impact-generated hydrothermal systems	Short-lived heat, fractured substrates, transient fluids	$\Psi_{\mathrm{cyc}}$, $\Xi_{\mathrm{pre}}$	Duration and preservation may dominate over initial energy.
Icy-ocean-world seafloors and plume sources	Hidden water–rock reactions beneath ice, uncertain transport to sample	$\Theta_{\mathrm{red}}$, uncertain $\Xi_{\mathrm{pre}}$	Geochemical activity and recoverable evidence can be strongly decoupled.

**Table 4:** Principal variables and symbols used in this study.

Symbol	Units or Domain	Meaning
$N_{s}$	integer	Number of chemical species in the modeled environment.
$N_{r}$	integer	Number of reactions in the modeled reaction set.
*R*	J mol^−1^ K^−1^	Universal gas constant.
*T*	K	Absolute temperature.
*P*	Pa	Pressure used in standard-state and activity calculations.
*F*	C mol^−1^	Faraday constant.
n	mol	Species amount vector; component $n_{i}$ is the amount of species *i*.
S	dimensionless	Stoichiometric matrix; entry $S_{ir}$ is positive for products and negative for reactants in reaction *r*.
J	mol s^−1^	Vector of control-volume net reaction fluxes, with component $J_{r}$.
$q_{\mathrm{in}}$, $q_{\mathrm{out}}$, $q_{\mathrm{phase}}$	mol s^−1^	Inflow, outflow, and phase-transfer terms in the open-system mass balance.
$\xi_{r}$	mol	Extent of reaction *r*.
$J_{r}$	mol s^−1^	Net reaction flux integrated over the chosen control volume for reaction *r*.
y	m	Spatial position vector within the control volume.
$\varrho_{r}\left(y,t\right)$	mol m^−3^ s^−1^	Local volumetric net reaction-rate density for reaction *r* at position y and time *t*.
$\mu_{i}$	J mol^−1^	Chemical potential of species *i*.
$a_{i}$	dimensionless	Chemical activity of species *i*.
$\Delta_{r}G$	J mol^−1^	Gibbs free energy change of reaction *r*.
$\Delta_{r}G^{\circ }$	J mol^−1^	Standard Gibbs free energy change of reaction *r* at specified *T* and *P*.
$A_{r}$	J mol^−1^	Chemical affinity of reaction *r*, defined as $A_{r}=-\Delta_{r}G$.
${\dot{\Sigma }}_{\mathrm{int}}$	J K^−1^ s^−1^	Internal entropy-production rate.
$P_{\mathrm{ex}}$	J s^−1^	Locally dissipative redox power potentially accessible to chemistry in the modeled control volume.
$\zeta_{\mathrm{cap}}$	[0, 1]	Optional coupling factor for the fraction of local redox power captured by a specified synthetic pathway.
${\left[u\right]}_{+}$	units of *u*	Positive-part operator, ${\left[u\right]}_{+}=\max\left(0,u\right)$.
V	m^3^	Control volume over which local reaction-rate densities are integrated.
$R_{\mathrm{red}}$	set	Subset of reactions treated as redox reactions in the modeled environment.
$\tau_{\mathrm{obs}}$	s	Observation, residence, or exposure timescale used in redox-exergy normalization.
$E_{\mathrm{ref}}$	J	Context-dependent reference energy for nondimensional redox-exergy mapping.
$z_{r}$	mol e^−^ mol^−1^	Electron stoichiometric number for redox reaction *r*.
$E_{r}$	V	Redox cell potential for reaction *r*.
$E_{r}^{\circ }$	V	Standard redox cell potential for reaction *r*.
$Q_{r}$	dimensionless	Reaction quotient for reaction *r*.
$N_{m}$	integer	Number of mineral classes included in the catalytic-interface term.
$N_{f}$	integer	Number of declared reaction families in the catalytic-interface term.
$A_{\mathrm{surf},m}$	m^2^	Reactive surface area of mineral class *m*.
$A_{\mathrm{ref}}$	m^2^	Reference reactive surface area used to nondimensionalize the catalytic-interface term.
$\kappa_{m,f}$	dimensionless	Reaction-family-conditioned catalytic efficacy assigned to mineral class *m* for reaction family *f*.
$\pi_{f}$	[0, 1]	Weight assigned to reaction family *f* in the catalytic-interface term; $\sum_{f}\pi_{f}=1$.
$\lambda_{2}$	dimensionless	Algebraic connectivity of the projected reaction graph.
$C_{\mathrm{prod}}$	set	Products reachable from feedstock species under the allowed reaction set.
$C_{\mathrm{auto}}$	set	Reachable products participating in catalytic or autocatalytic closure.
$\lambda_{\mathrm{ref}}$	dimensionless	Reference connectivity scale in the network term.
$\varepsilon_{\mathrm{card}}$	dimensionless	Small positive constant preventing division by zero in cardinality ratios.
$\tau_{\mathrm{env}}$	s	Characteristic environmental cycling time.
$\tau_{r}$	s	Characteristic reaction time for reaction *r*.
$D_{r}$	dimensionless	Damköhler-like number, $D_{r}=\tau_{\mathrm{env}}/\tau_{r}$.
$\sigma_{\mathrm{cyc}}$	dimensionless	Width parameter controlling tolerance around the optimal cycling condition $D_{r}=1$.
$C_{\mathrm{conc},r}$	[0, 1]	Concentration-retention factor for reaction *r* under environmental cycling.
$\alpha_{r}$	[0, 1]	Reaction-importance weight in the cycling score; $\sum_{r}\alpha_{r}=1$.
$\chi_{\mathrm{host}}$	[0, 1]	Suitability of the host mineral or phase for preserving the signal class.
$t_{\mathrm{arch}}$	s	Duration over which a signal must survive in the archive.
$k_{\mathrm{loss}}\left(t\right)$	s^−1^	Total time-dependent destruction rate for an archived signal.
$k_{\mathrm{ox}}$, $k_{\mathrm{hyd}}$, $k_{\mathrm{rad}}$, $k_{\mathrm{therm}}$, $k_{\mathrm{diag}}$	s^−1^	Oxidative, hydrolytic, radiolytic, thermal, and diagenetic loss-rate terms.
$\Theta_{\mathrm{red}}$	[0, 1]	Redox-exergy availability score.
$\Lambda_{\mathrm{cat}}$	[0, 1]	Catalytic-interface density score.
$\Omega_{\mathrm{net}}$	[0, 1]	Reaction-network closure and connectivity score.
$\Psi_{\mathrm{cyc}}$	[0, 1]	Environmental cycling efficacy score.
$\Xi_{\mathrm{pre}}$	[0, 1]	Preservation potential score.
$\Phi_{\mathrm{org}}$	[0, 1]	Prebiotic geochemical organization score excluding preservation.
$\Phi_{\mathrm{PGM}}$	[0, 1]	Geochemical Metabolic Potential used here as detectable proto-biosignature potential.
$X_{q}$	[0, 1]	Generic PGM component in the ordered component set, with q=1,…,5.
X	[0, 1]^5^	Full component vector $\left(\Theta_{\mathrm{red}},\Lambda_{\mathrm{cat}},\Omega_{\mathrm{net}},\Psi_{\mathrm{cyc}},\Xi_{\mathrm{pre}}\right)$.
$X_{\lim}$	[0, 1]	Limiting-component value, $X_{\lim}=\min_{q:w_{q}>0}X_{q}$.
$X_{\mathrm{thr}}$	[0, 1]	Declared weak-link reporting threshold for target-specific applications.
$f_{\mathrm{WL}}$	{0, 1}	Weak-link flag, set to unity when $X_{\lim}<X_{\mathrm{thr}}$.
${\tilde{w}}_{q}$	[0, 1]	Weight assigned to component $X_{q}$ when only the four organization terms are combined in $\Phi_{\mathrm{org}}$.
$Y_{q}$	$\left[\varepsilon_{\Phi },1\right]$	Regularized component $Y_{q}=\varepsilon_{\Phi }+\left(1-\varepsilon_{\Phi }\right)X_{q}$ used in Equation (23).
$w_{q}$	[0, 1]	Weight assigned to component $X_{q}$ in Equation (21); the weights sum to unity.
$\varepsilon_{\Phi }$	dimensionless	Small positive regularization constant used only to avoid logarithmic singularities in Equation (21).
$M_{\mathrm{sim}}$	integer	Number of synthetic environments in the parameter-space demonstration.
$x^{\left(s\right)}$	[0, 1]^5^	Component vector for synthetic environment *s*, ordered as $\left(\Theta_{\mathrm{red}},\Lambda_{\mathrm{cat}},\Omega_{\mathrm{net}},\Psi_{\mathrm{cyc}},\Xi_{\mathrm{pre}}\right)$.
$U_{\mathrm{org}}$	[0, 1]	Diagnostic organization coordinate, ${\left(\Theta_{\mathrm{red}}\Lambda_{\mathrm{cat}}\Omega_{\mathrm{net}}\right)}^{1/3}$; not a thermodynamic internal energy.
$V_{\mathrm{arc}}$	[0, 1]	Diagnostic archive-cycling coordinate, ${\left(\Psi_{\mathrm{cyc}}\Xi_{\mathrm{pre}}\right)}^{1/2}$; distinct from the control volume V.
d	data vector	Observational data used in evidence assessment.
$d_{X}$	contextual data vector	Data used to estimate the PGM component vector X before likelihood evaluation.
b	feature vector	Candidate proto-biosignature feature vector.
$C_{\mathrm{env}}$	contextual information	Environmental context used in Bayesian evidence assessment.
$H_{\mathrm{ab}}$	hypothesis	Low-organization abiotic null hypothesis.
$H_{\mathrm{pg}}$	hypothesis	Non-biological prebiotic geochemical-organization hypothesis generating the observed feature vector.
$H_{\mathrm{bio}}$	hypothesis	Biological hypothesis.
$K_{\mathrm{pg}/\mathrm{ab}}$	dimensionless	Bayes factor comparing $H_{\mathrm{pg}}$ with $H_{\mathrm{ab}}$.
$K_{\mathrm{bio}/\mathrm{ab}}$	dimensionless	Bayes factor comparing $H_{\mathrm{bio}}$ with $H_{\mathrm{ab}}$.
η	positive real	Sensitivity exponent linking $\Phi_{\mathrm{PGM}}$ to the context prior in Equation (36).
$\beta_{\mathrm{ab}}$	positive real	Baseline denominator in the illustrative prior-odds mapping of Equation (36).
$\gamma_{\mathrm{bio}}$	positive real	Independent baseline weight assigned to the biological hypothesis in Equation (37).
LDKB	text	Life Detection Knowledge Base.

**Table 5:** Operational mineralogical terminology used in the revised model. Examples are illustrative; a target-specific implementation must state which phases are actually present, reactive, and analytically resolved.

Term Used Here	Operational Meaning	Examples and Limits
Redox-sensitive mineral assemblage	Mineral association recording oxidation-state gradients, redox fronts, or coupled reduced and oxidized domains.	Fe-bearing oxides and spinels, Fe/Mn oxyhydroxides, sulfides, sulfates, native metals, alloys, and mixed-valence phases; interpretation requires texture and scale.
Catalytic mineral interface	Reactive surface accessible to fluids and relevant to a declared reaction family.	Clay surfaces, carbonates, silica, sulfides, oxides, native metals, alloys, phosphides, and mixed-valence phases; bulk abundance alone is insufficient.
Transition-metal catalytic phases	Metal-bearing phases for which electron transfer, adsorption, activation, or rate enhancement is specified.	Fe–Ni–S phases are one subset; Cu, Zn, Co, Ni, Fe, PGE-bearing phases, native metals, and alloys may matter when mechanisms are declared.
Redox-mineral-organic association	Spatially or stratigraphically coupled organics, redox-sensitive assemblages, and host phases.	A proto-biosignature-relevant context only if contamination, delivery, diagenesis, and abiotic synthesis are evaluated.

Abbreviation: PGE, platinum-group elements.

**Table 6:** Minimum reporting protocol for the reaction-network term $\Omega_{\mathrm{net}}$. The term is a screening descriptor and should be replaced by more detailed stoichiometric or kinetic analysis when data allow.

Step	Required Declaration	Purpose
Reaction set	Feedstocks, products, excluded reactions, and treatment of reversible pairs	Prevents arbitrary network boundaries and double counting.
Feasibility filter	$\Delta_{r}G$, activity model, kinetic threshold, transport renewal, and catalyst requirement	Removes edges that are topological but chemically implausible.
Graph projection	Species graph, hypergraph, bipartite graph, or stoichiometric matrix representation	Makes $\lambda_{2}$ and reachability comparable within a declared construction.
Edge weighting	Binary, flux-weighted, affinity-weighted, or confidence-weighted edges	Prevents low-flux or poorly constrained reactions from dominating closure.
Closure criterion	Definition of $C_{\mathrm{auto}}$, catalytic closure, or alternative autocatalytic core metric	Distinguishes molecular production from network persistence.
Uncertainty check	Sensitivity to reaction-set expansion, $\lambda_{\mathrm{ref}}$, and edge weights	Reports whether $\Omega_{\mathrm{net}}$ is robust or boundary-dependent.

**Table 7:** Operational guardrails for reporting and interpreting $\Phi_{\mathrm{PGM}}$.

Allowed Use	Prohibited Use
Report $\Phi_{\mathrm{PGM}}$ only with the component vector X, weights, $\varepsilon_{\Phi }$, uncertainty ranges, $X_{\lim}$, $X_{\mathrm{thr}}$, $f_{\mathrm{WL}}$, control volume, timescale, and signal class.	Report a scalar value alone, or compare values across targets with different signal classes, reference scales, weights, or measurement models.
Use $\Phi_{\mathrm{org}}$ to discuss prebiotic geochemical organization and $\Phi_{\mathrm{PGM}}$ to discuss recoverable proto-biosignature potential.	Treat $\Phi_{\mathrm{PGM}}$ as a probability of life, a probability of abiogenesis, a biosignature-confidence scale, or a universal mission-priority score.
Use the index to generate hypotheses, design measurements, select controls, and identify weak components requiring additional tests.	Use the index to infer biological activity, to rank real planets from synthetic anchors, or to bypass abiotic alternatives, contamination checks, and independent likelihood analysis.

**Table 8:** Minimum reporting standard for target-specific applications of $\Phi_{\mathrm{PGM}}$.

Component	Required Input	Normalization to Report	Required Control or Failure Check
$\Theta_{\mathrm{red}}$	Redox couples, activities, flux or residence-time model, *T*, *P*, pH/Eh convention	$E_{\mathrm{ref}}$, $\tau_{\mathrm{obs}}$, and reaction orientations	Low-flux or equilibrium redox control.
$\Lambda_{\mathrm{cat}}$	Mineral phases, reactive surface area, passivation, pH/T window, reaction families	$A_{\mathrm{ref}}$, $\kappa_{m,f}$, and $\pi_{f}$	Mineral blank, passivated surface, or non-catalytic phase.
$\Omega_{\mathrm{net}}$	Declared reaction set, feedstocks, feasibility filters, edge weights, closure criterion	$\lambda_{\mathrm{ref}}$, graph projection, $\varepsilon_{\mathrm{card}}$	Network-boundary sensitivity and flux/feasibility removal test.
$\Psi_{\mathrm{cyc}}$	Environmental recurrence, reaction times, concentration-retention factors	$\sigma_{\mathrm{cyc}}$, $\alpha_{r}$, $\tau_{\mathrm{env}}$, $\tau_{r}$	Non-cycling or degradation-dominated control.
$\Xi_{\mathrm{pre}}$	Signal class, host phase, degradation model, archive duration, access/sampling assumption	$\chi_{\mathrm{host}}$, $k_{\mathrm{loss}}\left(t\right)$, $t_{\mathrm{arch}}$	Alteration, radiation, contamination, or unpreserved-host control.

**Table 9:** Missing-data policy for target-specific applications.

Data State	Treatment	Required Report
Physically absent component	Set the corresponding $X_{q}$ to zero or to the declared physical lower bound.	State the limiting process and activate $f_{\mathrm{WL}}$ if $X_{q}<X_{\mathrm{thr}}$.
Observed but uncertain component	Treat $X_{q}$ as a distribution, not a point estimate.	Report interval, prior or likelihood source, and sensitivity of $\Phi_{\mathrm{org}}$ and $\Phi_{\mathrm{PGM}}$.
Latent or indirectly observed component	Marginalize over a declared model ensemble.	Report the latent variables, assumptions, and posterior-predictive uncertainty.
Not applicable to the declared signal class	Set the weight to zero before scoring or state that the scalar is not evaluated.	Report the reason for non-applicability and avoid comparison with implementations that include the component.
Same observation used in multiple roles	Use a joint likelihood or remove the overlap from one role.	Declare the partition between contextual data $d_{X}$ and candidate features b.

**Table 10:** Illustrative audit trail for a hypothetical Mars-analogue mudstone. Values are component intervals used to demonstrate how an application should be reported; they are not estimates for a real sample.

Component	Illustrative Interval	Possible Input Constraints	Main Uncertainty
$\Theta_{\mathrm{red}}$	0.35–0.65	Fe/S/P redox phases, alteration temperature, reaction affinity and residence time	Past flux continuity and activity corrections
$\Lambda_{\mathrm{cat}}$	0.40–0.70	Clay, phosphate, sulfide, oxide, and carbonate reactive surfaces	Reaction-family-specific rate enhancement
$\Omega_{\mathrm{net}}$	0.25–0.55	Reachable products and feasible redox-organic reaction paths	Incomplete reaction set and flux weighting
$\Psi_{\mathrm{cyc}}$	0.30–0.60	Wetting, burial, groundwater renewal, and alteration recurrence	Coupling between cycle time and reaction time
$\Xi_{\mathrm{pre}}$	0.65–0.90	Fine-grained host, shielding, mineral adsorption, and diagenetic history	Signal-class-specific loss rates

**Table 12:** Weak-link audit for the illustrative anchor scores in Table 11. The audit uses $X_{\mathrm{thr}}=0.25$ only as a declared example for this synthetic table; an empirical application must choose and justify its own threshold before scoring.

Anchor	Limiting Component(s)	$X_{\lim}$	$f_{\mathrm{WL}}$	Reporting Implication
Hadean hydrothermal	$\Xi_{\mathrm{pre}}$	0.50	0	Not weak-link flagged under the declared illustrative threshold.
Hadean wet–dry	$\Theta_{\mathrm{red}}$, $\Xi_{\mathrm{pre}}$	0.60	0	Not weak-link flagged; interpretation remains limited by redox and archive assumptions.
Noachian Mars	$\Omega_{\mathrm{net}}$, $\Psi_{\mathrm{cyc}}$	0.45	0	Not weak-link flagged; network and recurrence assumptions dominate uncertainty.
Enceladus-like	$\Xi_{\mathrm{pre}}$	0.25	0	Borderline archive limitation; not flagged because Equation (26) uses $X_{\lim}<X_{\mathrm{thr}}$.
Europa-like	$\Xi_{\mathrm{pre}}$	0.20	1	Weak-link constrained by preservation/recoverability in this illustrative scoring.
Rocky exoplanet	$\Xi_{\mathrm{pre}}$	0.45	0	Not weak-link flagged; geological marginalization remains the dominant interpretive issue.
Dry stagnant planet	$\Psi_{\mathrm{cyc}}$	0.10	1	Weak-link constrained by recurrence/cycling and low organization.

**Table 13:** Candidate proto-biosignature classes and their interpretation within PGM.

Class	Possible Observation	PGM Interpretation	Ambiguity and Control
Redox-mineral-organic association	Spatial association of organics with Fe, S, Mn, P, carbonates, clays, or sulfides	Coupling between energy gradients, mineral catalysis, and organic production or concentration	Abiotic redox fronts and diagenesis can mimic organization; require alteration controls
Molecular-complexity distribution	High-complexity molecules or non-random molecular ensembles in context	Possible selection-like production, concentration, or preservation within chemical networks	Abiotic complexification, contamination, and analytical bias; require blanks and abiotic mixtures
Catalytic mineral assemblage	Transition-metal sulfides, native metals/alloys, magnetite, mixed-valence oxides, clays, phosphates, carbonates, or silica in reactive interfaces	Enhanced $\Lambda_{\mathrm{cat}}$ and possible network coupling	Catalysis potential does not demonstrate that relevant reactions occurred; require reaction-path tests
Weak isotopic coherence	Small but spatially coherent C, S, Fe, N, or H isotope patterns	Possible sustained flux through redox pathways	Equilibrium fractionation, kinetic abiotic fractionation, and alteration; require spatial controls
Cyclic archive pattern	Repeated laminae, evaporitic sequences, fracture fillings, or hydrothermal pulses	Environmental recurrence consistent with $\Psi_{\mathrm{cyc}}>0$	Physical sedimentary cycles may be unrelated to prebiotic chemistry; require chemical coupling
Preservation-favorable host	Fine-grained sediments, silica, clays, sulfates, carbonates, phosphates	High $\Xi_{\mathrm{pre}}$ for organics or redox microtextures	Preservation potential can occur without prebiotic organization; require energy and network context

**Table 14:** Example data partitioning for a Mars-analogue mudstone. The same observation should not be used both to construct X and to supply an independent likelihood gain unless an explicit joint likelihood is used.

Data Class	Use in $d_{X}\to X$	Possible Use in *b* or Likelihood
Bulk redox-sensitive mineralogy	Estimate $\Theta_{\mathrm{red}}$ and feasible redox couples	Not independent unless spatial or isotopic pattern is withheld from X.
Catalytic mineral phases	Estimate $\Lambda_{\mathrm{cat}}$ and reaction-family plausibility	Independent only if surface-specific product association is separately measured.
Reachable reaction products	Estimate $\Omega_{\mathrm{net}}$ under a declared reaction set	Use as b only through joint likelihood if they defined the network.
Stratigraphic recurrence	Estimate $\Psi_{\mathrm{cyc}}$ and environmental timescale	May constrain likelihood if recurrence is measured independently from chemistry.
Organic/isotopic microstructure	Normally reserved for b after blanks and alteration controls	Can raise $K_{\mathrm{pg}/\mathrm{ab}}$ if not already used in component construction.

**Table 15:** Qualitative comparison of planetary regimes under PGM.

Regime	Likely High Terms	Likely Limiting Terms	Proto-Biosignature Implication
Hadean-Eoarchean Earth	$\Theta_{\mathrm{red}}$, $\Lambda_{\mathrm{cat}}$, $\Psi_{\mathrm{cyc}}$	Preservation of earliest signals	Multiple environments may have supplied complementary PGM components.
Noachian Mars	$\Xi_{\mathrm{pre}}$, local $\Lambda_{\mathrm{cat}}$, local $\Theta_{\mathrm{red}}$	Duration and continuity of flux; radiation exposure	Strong archival targets where organics, redox-sensitive mineral assemblages, and fine sediments co-occur.
Enceladus	$\Theta_{\mathrm{red}}$, plume access to ocean materials	Hidden archives; uncertain network closure	Active water–rock geochemical activity may be sampled, but biological inference remains difficult.
Europa	Potential $\Theta_{\mathrm{red}}$, possible $\Lambda_{\mathrm{cat}}$	Access, transport history, archive preservation	Surface and plume materials require linkage to ocean source regions.
Rocky exoplanets	Atmospheric disequilibrium proxies	Geological context, retrieval uncertainty, false positives	PGM acts as a prior connecting atmospheric data to planetary processes.

**Table 16:** Benchmark classes required for empirical calibration of PGM components.

Benchmark Class	Expected Behavior	Falsifying or Recalibrating Outcome
Low-organization abiotic controls	Low $\Phi_{\mathrm{org}}$, low structured organic/mineral co-localization	Frequent high proto-biosignature-like patterns without energy-catalysis-cycling overlap.
Prebiotic organized analogues	Intermediate to high $\Phi_{\mathrm{org}}$ with non-diagnostic but structured feature vectors	No enrichment in network closure or contextual molecular structure relative to null controls.
Biological or fossil-biological controls	High biological likelihood and independent biosignature indicators	PGM alone cannot replace biological evidence; it should not be tuned to force biological classification.
Alteration and contamination controls	Variable preservation with known overprinting or contamination history	Apparent proto-biosignatures disappear after blanks, spatial controls, or alteration modeling.
One-low-component stress tests	Suppression by the limiting component despite high values elsewhere	High $\Phi_{\mathrm{PGM}}$ when a required component is absent would require weight or mapping revision.

**Table 17:** Experimental and technical requirements for target-specific PGM implementation. Existing methods are listed only as examples; their applicability depends on sample return, in situ instrumentation, remote sensing, contamination control, and target environment.

PGM Term	Required Observable	Existing or Near-Term Methods	Current Limitation	Needed Development
$\Theta_{\mathrm{red}}$	Redox couples, oxidation states, activities, gradients, fluxes	electrochemistry, micro-XANES, Mössbauer, Raman, mass spectrometry, thermodynamic speciation	Activities and fluxes are often inferred rather than measured directly.	Co-registered redox speciation, permeability, fluid chemistry, and reaction-rate constraints.
$\Lambda_{\mathrm{cat}}$	Reactive mineral surface area and reaction-family-specific catalytic efficacy	XRD, Raman, SEM/TEM, BET surface area, microreactor experiments, kinetic assays	Mineral abundance is not reactive surface; catalytic efficacy is reaction-specific.	Mineral–organic–fluid microreactors across pH, temperature, passivation, grain size, and competing solutes.
$\Omega_{\mathrm{net}}$	Feasible reaction graph, accessible feedstocks, products, and closure	thermodynamic modeling, kinetic networks, mass spectrometry, chromatography, molecular-complexity metrics	Network closure is often latent because species inventories are incomplete.	Coupled thermodynamic–kinetic filtering with measured molecular inventories and uncertainty propagation.
$\Psi_{\mathrm{cyc}}$	Recurrence period, residence time, concentration-retention, reaction times	time-series experiments, stratigraphy, zoning, transport models, experimental cycling reactors	Ancient or remote recurrence histories are usually reconstructed indirectly.	Multiscale experiments and field analogues linking forcing period, chemistry, and preserved record.
$\Xi_{\mathrm{pre}}$	Host suitability, destruction rates, alteration, sampling accessibility	spectroscopy, microscopy, isotope analysis, radiolysis/oxidation tests, diagenetic modeling	Formation and preservation can be difficult to separate.	Paired mineralogical, textural, molecular, and contamination-controlled preservation experiments.

**Table 18:** Current barriers and development pathways for making PGM operational in laboratory, field, mission, and remote-sensing contexts.

Current Barrier	Development Pathway
Earth-centred calibration and limited planetary transferability	Build broader analogue suites with basaltic, ultramafic, evaporitic, icy-world, impact, and volcanic-arc compositions; report target-specific priors instead of universal defaults.
Scale dependence of redox and mineralogical heterogeneity	Couple micrometre-scale mineral/organic mapping with sample-, facies-, and field-scale upscaling rules; avoid bulk scores where microdomains control interpretation.
Incomplete redox inventories and activity constraints	Develop co-registered oxidation-state, mineralogical, fluid-chemistry, permeability, and reaction-rate measurements.
Uncertain catalytic efficacy of mineral phases	Run reaction-family-specific microreactor experiments on sulfides, native metals, alloys, oxides, phosphides, clays, carbonates, and mixed-valence phases under controlled passivation and fluid conditions.
Latent reaction-network closure	Combine molecular inventories with thermodynamic feasibility, kinetic filtering, graph analysis, and uncertainty-aware reaction-network modeling.
Formation-preservation ambiguity	Pair molecular detections with host mineralogy, texture, stratigraphy, alteration state, radiation history, and contamination controls.
Limited temporal information	Use experimental cycling, mineral zoning, stratigraphic repetition, transport models, and repeated measurements to infer recurrence and residence times.
Mission and remote-sensing limitations	Prioritize co-registered instruments and sampling strategies that jointly constrain redox state, mineral context, organics, texture, and preservation rather than one isolated marker.

## Data Availability

No empirical planetary data were generated or analyzed in this theoretical study. The source code, synthetic input tables, generated CSV summaries, weak-link audit outputs, negative-control and stress-test summaries, figure-generation script, figure files, requirements file, and runtime environment report used for the numerical demonstration are openly available in Zenodo at https://doi.org/10.5281/zenodo.21217714 (accessed on 5 August 2026). The same reproducibility files are also supplied as Supplementary Materials.

## Supplementary Materials

The following supporting information can be downloaded at https://www.mdpi.com/article/10.3390/life16081312/s1. The Supplementary Materials contains the Python script used to generate Figure 1, synthetic input and output tables, the weak-link audit, negative-control and stress-test summaries, figure files, a requirements file, and a runtime environment report. These files reproduce Figure 1 and the numerical summaries reported in Section 7.

## Abbreviations

The following abbreviations are used in this manuscript:

BET	Brunauer–Emmett–Teller
CoLD	Confidence of Life Detection
CSV	comma-separated values
LDKB	Life Detection Knowledge Base
PGE	platinum-group elements
PGM	planetary geochemical metabolism
$\Phi_{\mathrm{PGM}}$	nondimensional Geochemical Metabolic Potential defined in Equation (21)
SEM	scanning electron microscopy
TEM	transmission electron microscopy
XANES	X-ray absorption near-edge structure
XRD	X-ray diffraction
