# Supplementary material for: Proto-Biosignatures and Planetary Geochemical Metabolism: A Thermodynamic Screening Model of Prebiotic Geochemical Organization
Source: Life (Basel). 2026 Aug 10;16(8):1312. doi: 10.3390/life16081312 (PMC13514465; doi:10.3390/life16081312)
Supplement: Supplementary file 1 [file life-16-01312-s001.zip › life-4452067-supplementary/Figures/pgm_parameter_space.pdf]

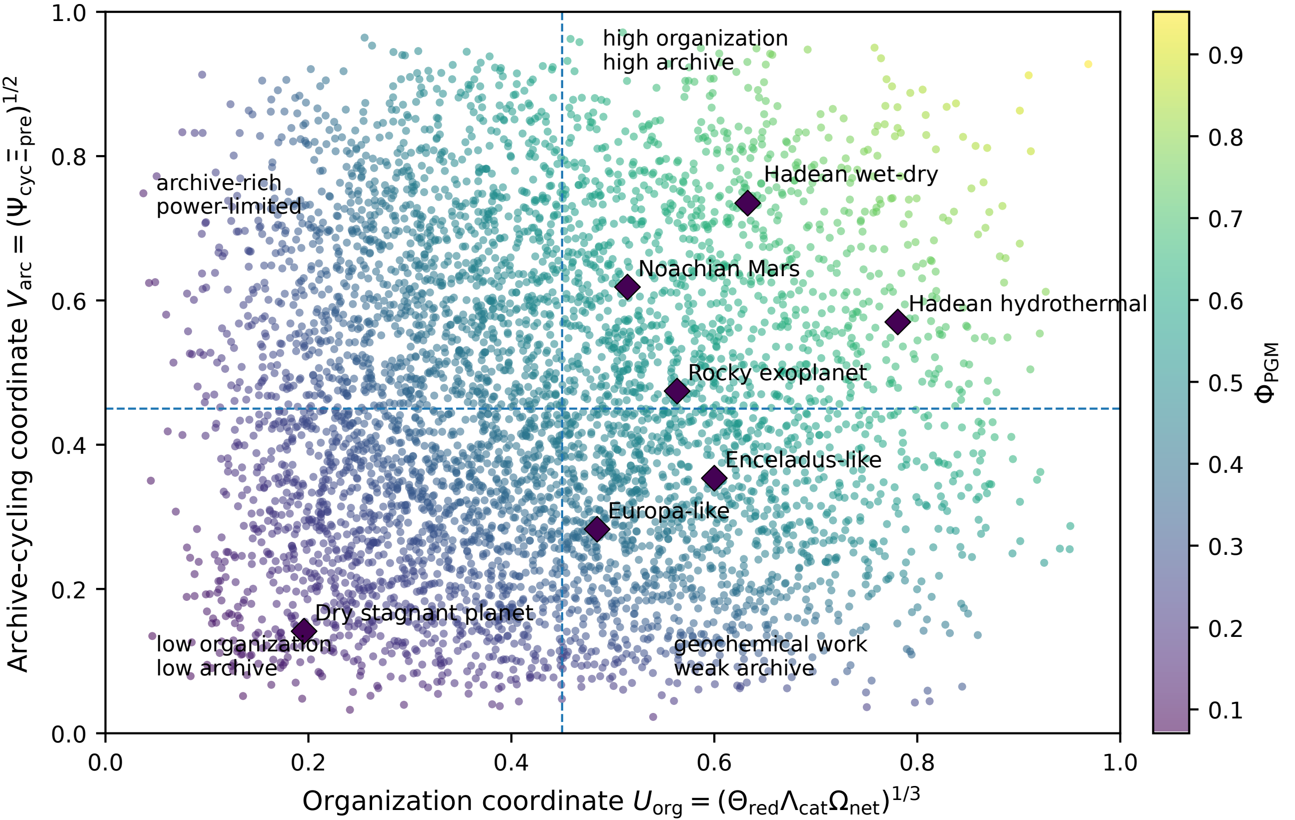

high organization
high archive
archive-rich
power-limited
low organization
low archive
geochemical work
weak archive
Hadean wet-dry
Noachian Mars
Hadean hydrothermal
Rocky exoplanet
Enceladus-like
Europa-like
Dry stagnant planet
Archive-cycling coordinate $V_{arc} = (\Psi_{cyc}\Xi_{pre})^{1/2}$
Organization coordinate $U_{org} = (\Theta_{red}\Lambda_{cat}\Omega_{net})^{1/3}$
Φ_{PGM}
